# Percolation of heterogeneous flows uncovers the bottlenecks of infrastructure networks

**DOI:** 10.1038/s41467-021-21483-y

**Published:** 2021-02-23

**Authors:** Homayoun Hamedmoghadam, Mahdi Jalili, Hai L. Vu, Lewi Stone

**Affiliations:** 1 Institute of Transport Studies, Faculty of Engineering, Monash University, Melbourne, VIC Australia; 2 Electrical and Biomedical Engineering, School of Engineering, RMIT University, Melbourne, VIC Australia; 3 Mathematical Sciences, School of Science, RMIT University, Melbourne, VIC Australia

**Keywords:** Civil engineering, Complex networks

## Abstract

Whether it be the passengers’ mobility demand in transportation systems, or the consumers’ energy demand in power grids, the primary purpose of many infrastructure networks is to best serve this flow demand. In reality, the volume of flow demand fluctuates unevenly across complex networks while simultaneously being hindered by some form of congestion or overload. Nevertheless, there is little known about how the heterogeneity of flow demand influences the network flow dynamics under congestion. To explore this, we introduce a percolation-based network analysis framework underpinned by flow heterogeneity. Thereby, we theoretically identify bottleneck links with guaranteed decisive impact on how flows are passed through the network. The effectiveness of the framework is demonstrated on large-scale real transportation networks, where mitigating the congestion on a small fraction of the links identified as bottlenecks results in a significant network improvement.

## Abstract (web summary)

Infrastructure networks are characterized by fluctuations of flow demand between different points and temporal congestion or overload on flow pathways. Hamedmoghadam et al. identify congestion bottlenecks in networks relevant to communication, transportation, water supply, and power distribution.

## Introduction

Recent theoretical advances in network science have considerably contributed to our understanding of complex systems, cutting across many disciplines from the social and technological sciences to the fields of ecology and biology^1–12^. In many modern studies, percolation theory^13^ has been frequently employed to characterize the structure, functionality, and resilience of network systems. In this approach, link failure is simulated by a percolation model which progressively removes links from the network^14,15^. The impact is usually measured via a reduction in the size of the network’s largest connected component, or giant component (GC), as links are gradually removed^16–18^. Different strategies for simulating link failures, e.g., random (error) or targeted (attack)^19^, make it possible to study a range of different topological characteristics.

In real infrastructure networks, however, pervasive phenomena such as various forms of congestion (e.g., traffic jams in transportation or packet congestion in communication networks) reduce the quality of flow movement on links in a continuous manner rather than necessarily causing a complete failure. To consider this, link-level flow dynamics on a network *G* can be modeled by associating each link *e*_*ij*_ (connecting node *i* to node *j*) with its own “quality” attribute *q*_*ij*_ ∈ (0, 1], which at any time indicates the link performance relative to an observed or predetermined maximum level of performance^20,21^. For example, in a road traffic network with the speed on each road changing temporally, link quality *q*_*ij*_ can be defined as the ratio of instantaneous traffic speed to the speed limit of the link *e*_*ij*_^22,23^, or in a communication network, quality can be defined as the instantaneous delivery rate of packets flowing along a link^24^.

**Fig. 1:**
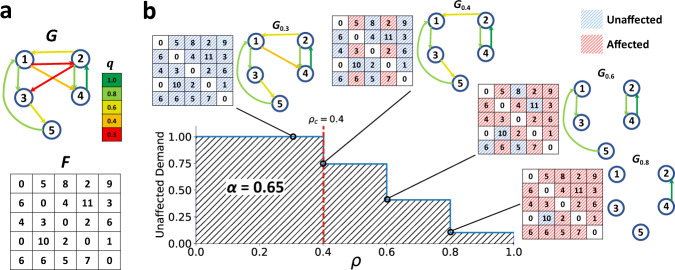
Percolation on an example demand-serving network. **a** Network *G* with size *n* = 5, where quality *q*_*ij*_ of each link *e*_*ij*_ is color-coded (according to the color-bar). Matrix *F* quantifies the flow demand between all pairs of nodes which sums up to 100 units in total. **b** The percolation process is simulated by increasing a threshold *ρ* while removing links *e*_*ij*_ with *q*_*ij*_ ≤ *ρ*. Subnetwork *G*_*ρ*_ is visualized at different *ρ*’s with its corresponding affected (red) and unaffected (blue) flow demand color-coded in matrix *F*. In this example, by definition, the system collapses at *ρ*_*c*_ = 0.4, when the 5-nodes strongly connected GC disintegrates into two strongly connected components of sizes 2 and 3, while unaffected demand (UD) is still at 75%. The reliability of the network *G* is *α* = 0.65, found by calculating the area under the curve of UD versus *ρ*.

Percolation models have been used to study the organization of link-qualities in networks^23,25,26^. The basic concept requires examining a single network *G* which may change in time, but at each particular time, the structure and link qualities represent the system’s state. The percolation process on *G* may be seen as a function of a threshold *ρ* where 0 ≤ *ρ* ≤ 1. For any specific threshold *ρ*, the idea is to delete any link in *G* with quality *q*_*ij*_ for which *q*_*ij*_ ≤ *ρ*, leaving the subnetwork *G*_*ρ*_; see the process on a small network in Fig. 1. We can then gain insights into the network *G*’s properties by monitoring the geometrical phase transitions in *G*_*ρ*_ as *ρ* varies from *ρ* = 0 to *ρ* = 1. (Note that the whole percolation process is performed on one network snapshot, thus the quality of links representing the state at that snapshot remain fixed during the process.)

Of special interest is the critical percolation threshold *ρ* = *ρ*_*c*_ at which the GC suddenly fragments into components of smaller size. The percolation threshold *ρ*_*c*_ is an informative measure of the global quality of network structure, indicating that the network fails to provide global connectivity only with paths of links having quality above *ρ*_*c*_^24,27,28^. While this generic critical phenomenon is of vital importance for characterizing networks, we will show that limiting attention exclusively to the GC and its sudden fragmentation reveals only a part of the full picture when studying real-world problems.

The primary goal in many critical infrastructure networks such as communication, power distribution, and water supply systems is to serve the demand for a certain amount of flow between each pair of nodes; we refer to such systems as “demand-serving networks.” In reality, the flow demand is often distributed heterogeneously over the origin–destination (O–D) node pairs in the network. For example, in transportation networks, the passenger travel demand is much larger between O–D points when one or both of them are hotspot locations^29^. The larger the flow demand between two nodes, the more crucial is their connecting paths^30^. When studying percolation in demand-serving networks, although the global connectivity is lost at percolation criticality, yet a substantial proportion of the network’s flow demand might be between O–D node pairs that remain connected in the subcritical phase. For example, if the bulk of the flow demand is contained within isolated small and medium-sized clusters (resulting from the GC fragmentation), the network can remain highly functional even after the GC collapse (see the example in Fig. 1b). In other words, the global dynamics in demand-serving networks is not only controlled by the structure and organization of link qualities, but also by the distribution of the flow demand.

The goal of the present paper is to add further realism to percolation-based network analysis by the inclusion of heterogeneous flow demand. We restrict our attention, first to real transportation networks as exemplary instances of demand-serving networks, but then demonstrate the generality of our proposed analysis. We introduce a theoretical framework to quantify the impact of each link’s quality (congestion) on flow movements through the network and use it to identify the network bottlenecks. We show that the percolation analysis suggested here can lead to different conclusions compared to those obtained solely from studying structural critical phenomena.

## Results

### The case of real infrastructure networks

We demonstrate the application of the proposed framework, on the bus and tram (on-road) public transportation (PT) systems in two major Australian cities, Melbourne and Brisbane, modeled using smart-card transaction data collected during September and October 2017 in Melbourne and over March 2013 in Brisbane. On-road PT systems are in constant conflict with road conditions, such as crowds, traffic, and signals, all negatively affecting the traveling flows by decelerating the PT vehicles. Separation of high demand O–D points by local pockets of congestion is an issue of considerable concern in transportation systems. The concept of travel demand distribution is fundamental to transportation theory^31^, but to date, has not been considered in percolation-based analysis of dynamical transportation networks.

We are first interested in the network representation of the transportation system (PT services with disregard to the passenger activity). In this respect, network *G*(*V*,*E*,*t*) at different times *t* of each particular day, was generated using the data time-stamped within the 2-h window centered at *t* (see “Methods” and Supplementary Note 1). Each node *i* ∈ *V* corresponds to a cluster of closely situated bus and tram stops. A directed link *e*_*ij*_ ∈ *E* connects its source node *i* to its target node *j*, if there is at least one PT service visiting node *i* and then *j* without any intermediate stops. A directed path from node *o* to node *d* is a sequence of links (all in the same direction) joining a sequence of distinct nodes, where the first node is *o* and the last node is *d*. In the second step, for each network *G*, the flow demand matrix *F* = [*f*_*od*_] was generated with *f*_*od*_ counting the number of passengers traveling from node *o* to node *d*, respectively, as the origin and destination points. Melbourne’s on-road PT network was comprised of approximately an average of 5500 (2800) nodes, 10,500 (4500) links, and a flow demand derived from a part of 470,000 (210,000) trips performed during a normal weekday (weekend day). Brisbane has a relatively smaller network with approximately 1400 nodes and 3400 links on average over a regular weekday.

**Fig. 2:**
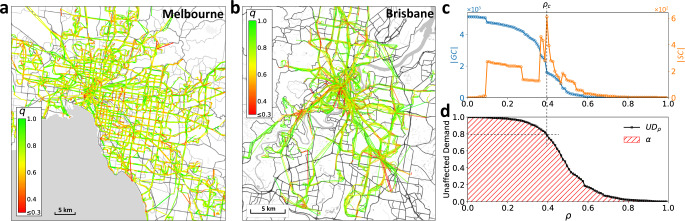
Real on-road public transportation (PT) networks. **a**, **b** The network representation of the PT system with color-coded link qualities *q* at *t* = 8 (8:00 A.M.), for Melbourne on 1 September 2017 (**a**), and for Brisbane on 1 March 2013 (**b**). **c** Percolation process on Melbourne’s network shown in (**a**). The size of the giant component ∣GC∣ and the size of the second-largest component ∣SC∣ are plotted as functions of the threshold *ρ*. The critical threshold *ρ* = *ρ*_*c*_ is determined as the point of maximal ∣SC∣ (vertical dashed gray line). **d** Percolation process on Melbourne’s network shown in (**a**). Unaffected demand is plotted as a function of *ρ* (UD_*ρ*_) which at percolation critical threshold shows the value of ${\rm{U{D}}}_{{\rho }_{c}}\approx 0.8$ (marked by dashed gray lines). The area hatched in red corresponds to the reliability *α* of the network in (**a**). Streetmap layers in **a** and **b** ©OpenStreetMap contributors^44^.

In order to quantify the link-level road conditions, we assign a quality attribute to each link *e*_*ij*_, calculated as

1 $${q}_{ij}(t)=\frac{\mathop{\min}\limits_{{t}^{\prime}}({\tau}_{ij}({t}^{\prime}))}{{\tau}_{ij}(t)},$$

where *τ*_*ij*_(*t*) is the travel time on the link *e*_*ij*_ at time *t* of the day. The quality attribute *q*_*ij*_(*t*) indicates the effect of temporal link-level congestion on flows passing through *e*_*ij*_. At any point in time, a high-quality link has relatively low travel time (or equivalently high velocity) compared to the rest of that day. In the following, for simplicity, we refer to the network and its attributes without the time parameter *t*. Figure 2a, b shows the spatial distribution of *q*_*ij*_ on the snapshot of the on-road PT network of Melbourne and Brisbane at 8:00 A.M. on a typical weekday. Note that the flow-demand matrix is determined from the passengers’ activity data, while the network *G* and its link qualities are determined from PT vehicles’ activity data.

The percolation process on a snapshot of Melbourne’s PT network is illustrated in Fig. 2c, indicating a percolation threshold of *ρ*_*c*_ = 0.39 when global connectivity is lost. However, as our analysis shows, over 80% of trips are between O–D node pairs that still remain connected even though *ρ* has reached the percolation threshold (when only the links with quality *q* > *ρ*_*c*_ are present). This highlights a problem with interpreting *ρ*_*c*_ as a reliability index (as per refs. ^26,27,32^) if the main interest is on heterogeneous passenger flow demand. This motivated us to develop a new approach to capture the reliability of heterogeneous demand-serving networks.

### Unaffected demand and network reliability

In this study, link removal in the percolation process should be viewed as a hypothetical procedure that unpacks the organization of congestion within a snapshot of the network in time. As explained before, the procedure is built upon constructing the subnetwork *G*_*ρ*_ which inherits all the links from the original network *G* except the most congested (lowest quality) links with qualities *q* ≤ *ρ*. By gradually increasing *ρ*, and at each step removing the shell of most congested links, the procedure extracts a series of subnetworks *G*_*ρ*_, each providing a different level of flow movement on the actual network. The impact of different levels of congestion on flows can then be examined by studying the properties of subnetworks *G*_*ρ*_, *ρ* ∈ (0,1].

Our approach is based on monitoring what we refer to as unaffected demand (UD), and requires keeping track of the flow-demand between all O–D node pairs during the percolation process. The network’s flow-demand is represented by the matrix *F* = [*f*_*od*_] of order *n* equal to the network size, where entry *f*_*od*_ is the amount of passenger-flow from origin node *o* to destination node *d* (see Fig. 1a). The matrix is normalized by dividing by the total demand ${{\mathbf{1}}}_{n}^{\text{T}\,}F{{\mathbf{1}}}_{n}$, to give $F/({{\mathbf{1}}}_{n}^{\text{T}}F{{\mathbf{1}}}_{n})$. (Here, **1**_*n*_ is a column vector of all *n* elements equal to one).

Using *F* that gives the flow-demand between any O–D pair, we can then calculate the UD as the percolation procedure proceeds and as low-quality links are removed. At any threshold *ρ*, the flow demand between an O–D pair is said to remain “unaffected” by link removals if there is at least one directed path from *o* to *d* remaining on *G*_*ρ*_. To assist in interpreting this, consider a link that is part of a path that begins from origin node *o* and reaches destination node *d*. When the link is removed (because it has fallen below threshold in quality), then the fraction of the demand ${f}_{od}/({{\mathbf{1}}}_{n}^{\text{T}}F{{\mathbf{1}}}_{n})$ remains unaffected by the link removal if and only if there is still at least one other directed path from *o* to *d*. We thus define UD_*ρ*_ as the fraction of the total flow between all the O–D pairs that remain unaffected at threshold *ρ* of the percolation process. In other words, UD_*ρ*_ is equal to the fraction of the demand on *G* that can travel between their O–D nodes without having to traverse any link with quality below the threshold *ρ*. See “Methods” for the formulation of UD_*ρ*_.

It is instructive to examine how UD_*ρ*_ varies with increasing *ρ* on the example network shown in Fig. 1a, where the total volume of flow demand is 100 by some arbitrary unit of measurement and UD_0_ = 100/100 initially. When *ρ* = 0.3 (Fig. 1b), two links of the lowest quality (colored red) are removed, but this does not affect the flow between any pair of nodes, and thus UD_0.3_ = 1. When *ρ* = 0.4, however, removal of the link 1 → 4 prevents flows from reaching nodes 2 or 4 from either node 1, 3, or 5, by any path on *G*_0.4_. The proportions of affected flows sum up to 25/100, thus the UD drops to UD_0.4_ = 0.75.

We now present our key index for assessing the reliability of demand-serving networks. We define the demand-serving reliability *α*, as the area under the curve of UD_*ρ*_ over the domain of *ρ* (hatched area under the curve in Fig. 1b). In compact form, this can be formulated as

2 $$\alpha = \int_{0}^{1}{\rm{UD}}_{\rho }d\rho = \int_{0}^{1}\frac{\,{\text{tr}}\,(R_{\rho }F^{\text{T}})}{{{\mathbf{1}}}_{n}^{\,{\text{T}}\,}F{{\mathbf{1}}}_{n}}d\rho ,$$

where tr(.) is the trace of the *n* × *n* square matrix. As seen in Eq. (2), it is also possible to formulate UD_*ρ*_, and as a result *α*, in simple mathematical terms making use of the network’s so-called reachability matrix *R* and the flow demand matrix *F* (see “Methods”).

The meaning of UD_*ρ*_ and *α*, becomes clearer from viewing plots as in Fig. 2d. In such plots, if UD_*ρ*_ rapidly drops at relatively low *ρ* values, then most of the flow demand is constrained to traverse low-quality (congested) links. This in turn lowers the area under the curve of UD_*ρ*_, and the reliability *α* will consequently be low. If UD_*ρ*_ does not drop rapidly until much larger *ρ* values, then most of the demand is between node pairs that are connected via paths of high-quality links, and the reliability *α* will be high. Hence, reliability *α* gives an indication of how well the flows pass between their O–D points given the organization of congestion on the network. (See Supplementary Note 2 on the relevance of the links’ flow-capacity to our reliability analysis.)

Let ∣GC_*ρ*_∣ be the size (number of nodes) of the GC in *G*_*ρ*_. In the “Methods”, we show that when flow demand distribution is homogeneous (i.e., the passenger flow *f*_*od*_ is the same between all reachable pairs of nodes *o* and *d*), then on any large-enough undirected network, we have $| {\rm{GC}}_{\rho }| \approx n.\sqrt{{\rm{UD}}_{\rho }}$ at any threshold *ρ* during the percolation. Thus, only by assuming a uniform flow demand over the network, UD is able to replicate the percolation analysis based on monitoring the GC; this is also confirmed numerically later in the paper. Second, with heterogeneous flow demand, the above relation no longer holds, and the fall-off of UD as a function of *ρ* provides its unique description of the system dynamics. By aggregating UD’s description of the system, *α* provides a simple and useful indication of network reliability.

### Bottleneck identification

Improving the infrastructure networks via protection or enhancement of a minimal set of links is currently receiving intense research interest^20,33,34^. Our framework suggests a new approach for identifying network bottlenecks. Here, inspired by the work on the maximum capacity paths problem^35^, we introduce the link criticality score *s*_*ij*_, which quantifies the overall role of each link *e*_*ij*_ in impeding the network flows.

**Fig. 3:**
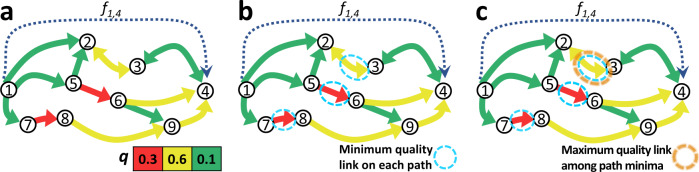
Finding the limiting link between an origin-destination node pair. **a** A small network with color-coded link qualities *q*, where as an example, we demonstrate the process to identify the limiting link between the O–D node pair (1,4) having a directed flow demand of *f*_1,4_. **b** The available paths from node 1 to node 4 (and path’s minimum-quality link) are 1 → 2 → 3 → 4 (*e*_2,3_), 1 → 5 → 2 → 3 → 4 (*e*_2,3_), 1 → 5 → 6 → 4 (*e*_5,6_), 1 → 5 → 6 → 9 → 4 (*e*_5,6_), 1 → 7 → 8 → 9 → 4 (*e*_7,8_). **c** Among the minimum-quality links on these paths, *e*_2,3_ has the maximum quality. Just below the threshold *ρ* = 0.6, still, two paths connect node 1 to node 4, but then with *e*_2,3_ removed, node 4 becomes unreachable from node 1 on *G*_0.6_. The limiting link associated with node pair (1,4) is *e*_2,3_, thus, an increase in *q*_2,3_ will increase the lowest quality that the flow from node 1 to node 4 is constrained to interfere with. The ratio of *f*_1,4_ to the total demand, is added to criticality score *s*_2,3_ of the link *e*_2,3_ to reflect the importance of its quality *q*_2,3_ for flow movement over the network.

Suppose there is a set of different directed paths Ψ_*od*_ that connect node *o* to node *d* (see Fig. 3a). On each path *ψ* ∈ Ψ_*od*_, we search for the link with the minimum quality (Fig. 3b). Among those particular links, we choose the link with the maximum quality (Fig. 3c), denote it by ${e}_{od}^{* }$, and refer to it as the “limiting link” associated with the O–D node pair (*o*,*d*). For simplicity, let us assume that each link quality value on the network is unique. Then, there will be only a single limiting link between any reachable pair of nodes. For a link *e*_*ij*_, if it is never found to be the limiting link between a node pair, it will have a criticality score of zero. If ${e}_{ij}={e}_{od}^{* }$ for only a single pair (*o*,*d*), then the link criticality score *s*_*ij*_ will be the fraction of the total demand that flows from *o* to *d*, i.e.,

3 $${s}_{ij}=\frac{{f}_{od}}{{{\mathbf{1}}}_{n}^{\text{T}}F{{\mathbf{1}}}_{n}}.$$

The index relies on the feature that, for a given O–D pair, during the hypothetical percolation process, as soon as the threshold *ρ* reaches the quality of the associated limiting link, removal of the latter causes complete rupture of all paths between the O-D pair on *G*_*ρ*_. This means the limiting link has the lowest quality, that flows are constrained to traverse in order to travel between their origin and destination nodes on the actual network *G*. If the link *e*_*ij*_ is the limiting link between several node pairs (see Supplementary Fig. 3A), Eq. (3) extends to

4 $${s}_{ij}=\sum _{o,d\in V,{e}_{od}^{* }={e}_{ij}}\frac{{f}_{od}}{{{\mathbf{1}}}_{n}^{\text{T}}F{{\mathbf{1}}}_{n}}.$$

We have identified an important relationship that connects the link quality (*q*_*ij*_), the link criticality score (*s*_*ij*_), and the network reliability (*α*), namely

5 $$\sum _{{e}_{ij}\in E}{s}_{ij}.{q}_{ij}=\alpha ,$$

as proven in “Methods” (and illustrated in Supplementary Fig. 3B). It can be rigorously shown that for any link *e*_*ij*_, increasing *q*_*ij*_ within a non-empty range will increase the network reliability *α*, with the magnitude of increase being proportional to *s*_*ij*_ (see Supplementary Note 3). (This is a nontrivial problem since alteration of the quality of any link in the network can change the criticality score of multiple links.) Therefore, after ranking the links according to their criticality scores, a desired number of the top-ranked links can be identified as network bottlenecks.

Numerical simulations were used to test how accurately the ranking of links based on link criticality scores (CS ranking) can identify network bottlenecks. To this end, first, a simple intuitive method was used to find the true bottleneck links, i.e., the ground truth. The method requires perturbing the quality *q*_*ij*_ of individual links by a small positive amount *ε* (we chose this to be *ε* = 0.01), one by one, and then ranking the links according to their ability to perturb the reliability score *α*. The link whose perturbation increases the reliability *α* the most is deemed to be the most critical link etc. Through this brute-force procedure, the true ranking (TR) of the criticality of all links are obtainable.

We applied the ranking schemes on random geometric graphs (RGGs) with *n* = 100 nodes spread over the space [0,10]^2^ uniformly at random, and links connecting any pair of nodes with distance less than *r*_0_ = 1.5 (which ensures connectivity and having over 300 links^36^).

**Fig. 4:**
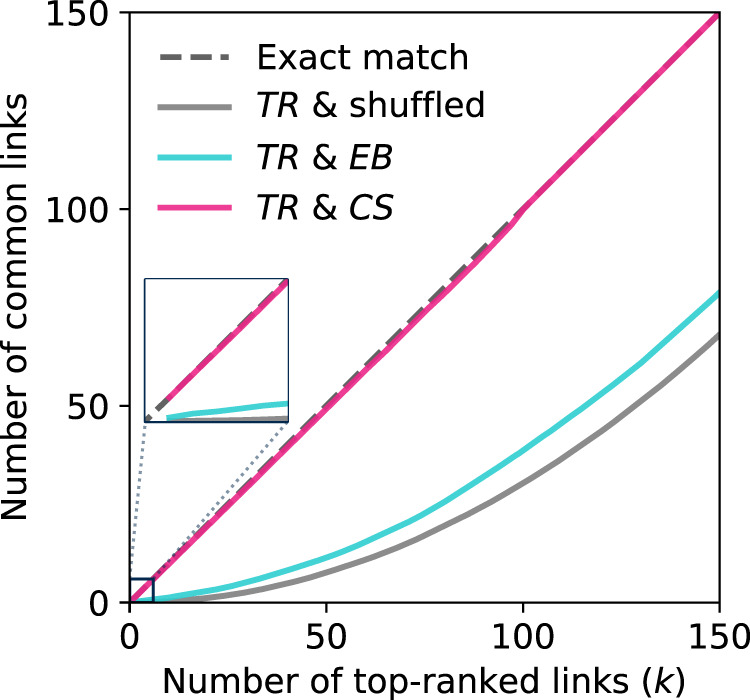
Assessing the accuracy of link criticality score index in identifying the true bottlenecks. The true ranking of links (TR) in terms of their improvement effect on network reliability *α*, is compared to rankings based on link criticality score (CS), edge betweenness centrality (EB), and randomly shuffled rankings of links. Each curve shows the number of common links between the set of top-*k* true bottlenecks and top-*k* bottlenecks of another ranking scheme. A ranking equal to TR leads to a line lying on the diagonal dashed line.

To compare CS and TR rankings, we took the set of *k* top-ranked links in each ranking and counted the number of common links between them. Figure 4 shows the number of common links between the CS and true top-bottlenecks of the network for *k* = 1, 2, …, 150, averaged over 500 realizations. We also compared against the ranking obtained by the conventional index edge betweenness centrality^37^ (EB), and a randomly shuffled ranking. The set of CS bottlenecks was found to be almost exactly the same as the set of true bottlenecks (TR) with (on average) 98–100% of their elements matching for different *k* values. The EB and the shuffled rankings were by far inferior to the CS scheme as Fig. 4 shows, although as might be expected, the EB ranking had a higher accuracy compared to the shuffled ranking. Note that unlike the brute-force approach used to find TR, the criticality score *s* of all network links can be calculated via scalable algorithms, e.g., our suggested modified Dijkstra’s algorithm (see Supplementary Note 3).

### Application to public transportation networks

We return now to using the above tools to study the PT networks of Melbourne and Brisbane. Figure 2c illustrates the percolation process on Melbourne’s bus and tram (on-road) PT network (at 8:00 A.M. on 1 September 2017) through ∣GC∣ and the size of the second-largest component (∣SC∣) as functions of *ρ*. In practice, the percolation threshold is determined as the threshold *ρ* = *ρ*_*c*_ at which ∣SC∣ is maximal^38^. In Fig. 2c, the point of maximal ∣SC∣ captures the GC collapse, however, this was not always the case at other times and dates. The GC fragmentation during the percolation process was often blurred out rather than demonstrating a drastic change in ∣GC∣, or in other cases, appeared as multiple peaks in ∣SC∣ which makes it difficult (if not impossible) to identify the critical threshold (Supplementary Fig. 4); ref. ^39^ reports similar observations in the road network of multiple cities. The index *α* evaluates the network according to the whole percolation process and does not depend on the existence of a clear phase transition, making the above issue irrelevant.

Figure 2d demonstrates the percolation process shown in Fig. 2c, but this time with UD as a function of *ρ*. As pointed out before, at the critical percolation threshold *ρ*_*c*_ = 0.39 where the global connectivity on *G*_*ρ*_ breaks down, we see that UD_0.39_ = 0.8. Thus, 80% of all the trips on the network *G* are between O–D node pairs that remain connected after the breakdown of the GC, and only via paths of links with *q* > 0.39. This empirically demonstrates how characterizing a network based on *ρ*_*c*_ alone can be misleading when flow demand distribution is heterogeneous. In effect, during the percolation process, UD does not necessarily decline with the same rate as pairwise connectivity (see Supplementary Note 4 and Supplementary Fig. 5). For Melbourne’s PT network, the number of connected node pairs on *G*_*ρ*_ decreases faster than UD_*ρ*_, meaning that demand is higher within clusters of high-quality links in the network.

**Fig. 5:**
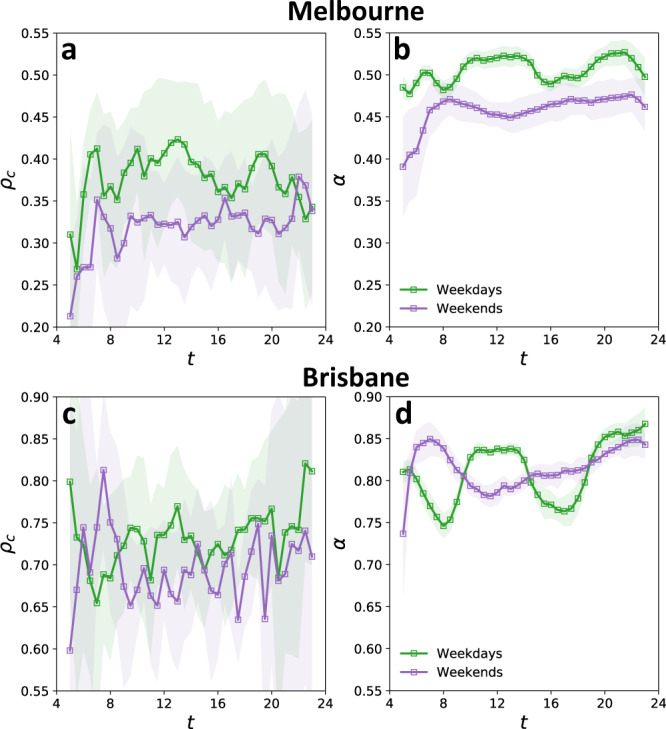
Reliability of the on-road public transportation (PT) networks. **a**, **b** Temporal evolution of *ρ*_*c*_ (**a**) and *α* (**b**) for Melbourne’s PT network, during weekdays (green) and weekends (purple). At each time *t*, curves show the mean, and shaded areas indicate the standard deviation of values around the mean, over September and October 2017. **c**, **d** Temporal evolution of *ρ*_*c*_ (**c**) and *α* (**d**) for Brisbane’s network averaged over the days in March 2013, separately for weekdays and weekends.

We also examined both reliability *α* and *ρ*_*c*_ on Melbourne’s (Brisbane’s) PT network over the main functioning hours of the system during September and October 2017 (March 2013), separately for weekdays and weekends. Temporally, *ρ*_*c*_ had relatively large fluctuations over the day, and there appeared to be no repeating pattern on a day to day comparison (see Fig. 5a, c for Melbourne and Brisbane networks, respectively). In contrast, the proposed reliability measure *α* followed a clear daily pattern (see Fig. 5b for Melbourne and Fig. 5d for Brisbane’s PT network) with variations that have a relatively small standard deviation. (Supplementary Note 5 and Supplementary Fig. 6A, C provide more details concerning *ρ*_*c*_ and *α* and their comparison.) The approximately 10% drops in *α* at 8:00 and between 16:00 and 18:00 are associated with weekdays’ morning and evening peak commuting periods when high rates of congestion and large numbers of commuters predictably increase the conflict between PT system and road conditions. Consistency of the daily evolution of *α* (for both Melbourne and Brisbane networks) with the circadian rhythm of urban human mobility and its low variability over different days indicate its success in unraveling the repeating daily pattern in complex interactions between major constituents of the system, namely, supply network structure, link-level congestion, and passenger flow demand (see Supplementary Note 5 for more detail). The results also suggest that Melbourne’s PT network is relatively stable over a day, despite multiple periods of intense traffic, which is partially due to more available PT services during the rush hours which increase the number of links and thus network density (see Supplementary Fig. 7).

Despite the larger flow demand and more extensive congestions during weekdays, *α* was larger for weekdays compared to weekends in Melbourne (Fig. 5b). This is because Melbourne’s PT network is fine-tuned for weekday demand, operating with a higher number of services during weekdays as compared to weekends. The larger number of PT services not only resulted in a larger number of network links but also led to a significantly higher link density during weekdays when compared to weekends (see Supplementary Fig. 7B). Higher link density of the network on weekdays means the availability of more paths between nodes and that if a path between two nodes includes congested links, it is generally more likely that an alternative less congested path exists. We also observed that in Melbourne’s PT network during weekends a significantly larger proportion of the trips are to/from the central business district (CBD) area, where the links are often subject to a higher level of congestion than elsewhere in the network. Lower link density of the network together with the large proportion of the passengers traveling to/from CBD on weekends, results in more conflict between flows and congestion (that is what *α* measures) which is reflected with the lower network reliability *α* during weekends. (From UD’s perspective, a larger proportion of the network demand has to pass through lower-quality links during weekends compared to weekdays.) In Brisbane, however, although the network has more links during weekdays, links (PT services) are supplying the transportation between a larger number of nodes, which keeps the link density of the network approximately the same between weekdays and weekends. As a result, unlike Melbourne, *α* fluctuated within approximately the same range during both weekdays and weekends for Brisbane’s PT network (Fig. 5d). Yet, similar to the case of Melbourne’s PT network, the daily evolution of Brisbane’s PT network reliability *α* on weekdays had distinct patterns from that of weekends.

### Bottlenecks of real transportation networks

Link criticality scores vary over time in temporal on-road PT networks. Therefore, we calculated the mean criticality score of each link over the course of the available data, and identified the network bottleneck links as those with the largest mean criticality scores, separately for weekdays and weekends. The identified bottlenecks were found to be robust, appearing with high criticality scores on most days (Supplementary Fig. 8).

**Fig. 6:**
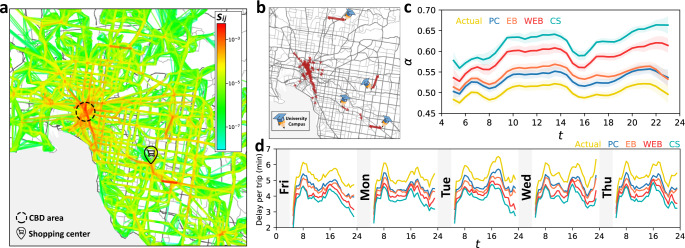
Bottleneck identification and amelioration on a real-world network. **a** Spatial distribution of the link criticality scores *s*_*ij*_ over Melbourne’s public transportation (PT) network during weekdays. The central business district (CBD) and the biggest shopping center in Melbourne are pinned on the map. **b** Top 100 weekday bottlenecks of Melbourne’s PT network, identified based on link criticality scores. Major university campuses outside Melbourne’s CBD area are pinned on the map. **c**, **d** The impact of ameliorating perturbations on bottlenecks identified by different approaches, i.e., criticality score (CS), edge betweenness centrality (EB), demand-weighted edge betweenness centrality (WEB), and percolation criticality (PC). The number of identified bottlenecks by each approach is equal to 2% of the average number of links that appear on the network. **c** Daily evolution of *α* calculated for the actual (yellow) and ameliorated networks associated with different bottleneck identification approaches. Results show the average (solid line) and standard deviation (shaded area) over the weekdays of September and October 2017. **d** Delay per trip (in minutes) caused by road congestions, on the actual and improved networks. Streetmap layers in **a** and **b** ©OpenStreetMap contributors^44^.

The spatial distribution of link criticality scores over Melbourne’s weekday PT network is portrayed in Fig. 6a (see also Supplementary Fig. 9A for Melbourne’s weekends and Supplementary Fig. 10A for Brisbane). Pockets of traffic congestions and crowds, which decrease the quality of PT network links, are usually formed around the high-demand urban hotspots. As a result, links with large criticality scores were found to be situated in urban hotspots and the areas surrounding them, making the spatial distribution of link criticality scores in surprising alignment with the urban morphology. Specifically, Melbourne’s biggest urban shopping center was surrounded by links with high criticality scores, and the top bottlenecks were mostly distributed around the single most significant hotspot of Melbourne which is the CBD. Furthermore, universities are good examples of urban hotspots that are only fully active on weekdays. Among the top bottlenecks of Melbourne’s network, we observed links to and from major universities (Fig. 6b) emerging only on weekdays (see Supplementary Fig. 9B). Given that the proposed method does not incorporate any geospatial information from the network, the surprising alignment between the locations pinned by identified bottlenecks and the urban hotspots, suggests that the method is capturing the actuality.

We also observed that four out of the top ten pain points on Melbourne’s road network reported in the media^40^ are overlapping with or in very close proximity to our identified top bottlenecks at morning rush hour. Since almost half of the reported ten points do not have bus or tram services in conflict with the road conditions, the results suggest that our methodology does indeed work well.

### Bottleneck amelioration

It is interesting to compare the effectiveness of our proposed CS-based bottleneck identification scheme, to other well-established bottleneck identification schemes. In particular, we compare against the bottlenecks identified based on the widely used edge betweenness (EB) centrality measure, here referred to as EB bottlenecks. We also use an extended version of the EB scheme, which incorporates the demand distribution by weighting the O–D node pairs when calculating the EB centrality of links, here referred to as Weighted EB or simply WEB. Alternatively, bottlenecks can be identified among the links removed at percolation criticality as used in ref. ^26^, which we refer to as PC bottlenecks. These bottlenecks termed “red bonds” in percolation theory^41^, glue the GC together by connecting the communities of higher-quality links. (For a more detailed description of the above approaches, see Supplementary Note 6.)

To compare these approaches, we separately ameliorated the bottlenecks of each type and monitored the response of the network in terms of changes to the demand-serving reliability *α*. In practice, the most obvious proposal for enhancing the reliability of an on-road PT network is to reduce the conflict of PT vehicles with road conditions at network bottlenecks, which can be achieved, for example, by giving signal priority to PT vehicles or allocating segregated (exclusive) PT lanes. Here, the bottlenecks are taken to be the top 2% most critical links in the network over time, according to each approach. Let *B* denote the set of bottlenecks identified by one of the schemes. We ameliorated the bottlenecks by synthetically increasing the qualities of bottleneck links *e*_*ij*_ ∈ *B*, to unity (*q*_*ij*_ = 1). Figure 6c (Supplementary Fig. 9A) compares the impact of ameliorating the bottlenecks identified by the four different approaches, as functions of time during weekdays (weekends) in Melbourne; see Supplementary Fig. 10B for Brisbane’s PT network. Amelioration of the CS bottlenecks resulted in more than 23% (26%) improvement in reliability *α* of Melbourne’s PT network, on average during weekdays (weekends). However, on average over both weekdays and weekends, amelioration of PC, EB, and WEB bottlenecks, only increased *α* by approximately 16%, 8%, and 6%, respectively. See Supplementary Fig. 10B, C for comparison between the effectiveness of different types of bottlenecks for Brisbane’s PT network.

The investigation was extended by verifying the impact of bottleneck amelioration on reducing the delay in passenger travel times. In order to calculate the delay caused by congestion, we first generated a congestion-free copy of the network at each time of a day by synthetically changing the actual travel time on each link to the minimum travel time observed on that link during the day. We assumed that each trip took place on the directed path with the minimum sum of the link travel times, between its origin and destination nodes. Then, for any particular network, the total delay was calculated as the absolute difference between the total travel time on the actual and the congestion-free copy of the network. Delay indicates the extent of the impeding effect of link congestions on passenger trips.

Separately for weekdays and weekends, we simulated the amelioration of the top CS, EB, WEB, and PC bottlenecks (the top 2% most critical links based on each scheme) of Melbourne’s PT network. The delay per passenger trip of 5.3 min (5.7 min) decreased to 3.8 min (4.2 min) by ameliorating the CS bottlenecks of weekdays (weekends). Figure 6d shows the delay per passenger trip on the actual and ameliorated networks at different times during the first five weekdays of September 2017; Supplementary Fig. 11B extends the results to two months of data. The time saved by amelioration of CS bottlenecks was 25% more than that of WEB bottlenecks while it was twofold compared to those of EB and PC bottlenecks. Ameliorating the top CS bottlenecks saved close to 2,000 hours of passenger travel time during a single morning peak period (7:00–9:00 A.M.), and approximately 11,000 hours of passenger travel time over a normal weekday.

### The generality of the proposed framework

**Fig. 7:**
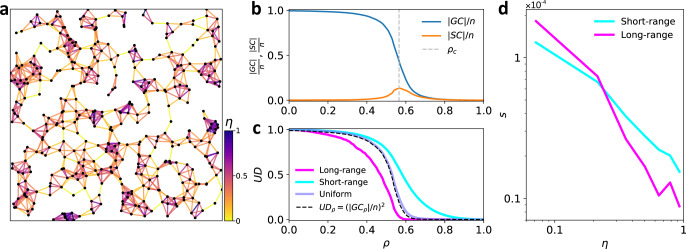
Capturing true properties of the demand-serving networks. **a** A sample RGG of size *n* = 400 with color-coded link overlap index *η*. **b** Normalized ∣GC∣ and ∣SC∣ during the percolation process averaged over 100 realizations of RGG structure with *n* = 2500 nodes and random link qualities. **c** Unaffected demand (UD) versus *ρ* for different flow demand scenarios on the RGG structure, averaged over 100 realizations. As predicted, the evolution of the GC size during the percolation process is approximately equal to $n.\sqrt{{\rm{UD}}_{\rho }}$ (see Methods) when the flow demand is uniform, which is the reason for the similarity between the blue and the dashed black curves. **d** Link criticality score *s* versus link overlap *η*, compared for short-range and long-range flow demand scenarios. See Supplementary Note 7 for extension of this analysis to the square grid and random graph structures.

In order to emphasize the generality of the proposed framework, we used undirected RGGs as a generic proxy of spatial networks and showed that the framework is able to reflect the true global flow-properties of the network. Here, RGG structures were generated by first distributing *n* = 2500 nodes uniformly at random on the plane ${[0,\sqrt{n}]}^{2}$, and then connecting any pair of nodes with Euclidean distance below *r*_0_ = 1.6. We chose *r*_0_ to be greater than the threshold ${r}_{0}^{c}\approx \sqrt{\mathrm{ln}\,(n)/\pi }\approx 1.58$ for which it is known^42^ that the network will be a.a.s. connected. The quality of each link was drawn uniformly at random from (0,1], making percolation a random link removal process depending only on the network topology. RGGs are built of clusters with high intra-connectivity, glued together by bridging links (Fig. 7a). This structure demonstrates a clear phase transition during the percolation process, as removal of a sufficient number of intercluster links causes an abrupt fragmentation of the GC (Fig. 7b).

Over each RGG instance, we distributed a fixed volume of flow demand, according to three different scenarios, namely, uniform, short-range, and long-range. In the uniform demand scenario, the total flow demand volume was divided equally among all reachable (*o*,*d*) node pairs; i.e., all entries of *F*, which correspond to a reachable node pair, are equal to a constant. Let *D*_*od*_ be the Euclidean distance between nodes *o* and *d*, and *D*_max_ the distance between the most distant node pair in the network. Then, to generate the short-range (long-range) flow demand scenarios, we picked a node pair (*o*,*d*) uniformly at random and then with probability $0.2{e}^{-0.2{D}_{od}}$ ($0.2{e}^{-0.2({D}_{\rm{max}}-{D}_{od})}$) added one unit to the volume of flow demand between that O–D pair *f*_*od*_, and repeated this until the fixed total flow volume was completely allocated to the node pairs over the network.

We simulated the percolation on 100 realizations of RGG structure for each one of the above flow demand distributions. During the percolation, we monitored the GC and SC, which are independent of the demand distribution, and also monitored the UD for different demand distribution scenarios (Fig. 7b, c). Remarkably, in Fig. 7c for the case of uniform flow, the percolation diagram as a function of *ρ* is the same for UD as it is for the square of ∣GC∣ (normalized by the network size). Thus, simulation results confirm the previously discussed theoretical relationship ${\rm{UD}}_{\rho }\approx {(| {\rm{GC}}_{\rho }| /n)}^{2}$ between evolution of the GC and UD when demand is uniformly distributed over the network. This shows that by assuming a uniform flow demand over the network, our method can provide an analogous analysis to that of monitoring the GC. Furthermore, UD shows logical sensitivity to the nonuniformity of flow demand distributions over the network. Long-range flows are more likely to get caught up in lower-quality links because each time they have to pass between clusters their choices become limited to a few bridging links. This resulted in lower reliability (*α* = 0.43) compared to when the flow-demand is uniformly distributed (*α*=0.50). In contrast, short-range flows are more likely to stay within the well-connected clusters of RGG, where there are more alternative paths available to bypass low-quality links. Hence, the network is more reliable for a short-range flow demand, which was fairly characterized by a higher *α*( = 0.58).

Here, we use RGG networks with different flow demand scenarios to verify the success of link criticality score in identifying network bottleneck links. We use the link overlap *η* ∈ [0,1] to determine whether a link belongs to a community (high overlap) or acts as an intercommunity bridge (low overlap); overlap of a link *e*_*ij*_ is defined as ${\eta }_{ij}=\frac{| {{\Gamma }}(i)\cap {{\Gamma }}(j)| }{| {{\Gamma }}(i)\cup {{\Gamma }}(j)| -2}$ where Γ(*i*) is the neighborhood set of node *i*. In Fig. 7a, links are color-coded according to their overlap index. The criticality score of intra-community (high overlap) links was found to be higher for the short-range flow demand scenario compared to the long-range scenario (Fig. 7d). This is consistent with the fact that short-range flows are more likely to have their origin and destination within a community, which makes the flow-carrying role of intra-community links more critical. Inter-community (low overlap) links have a stronger role in bridging between the remote points of the network, thus, the larger the proportion of the demand flowing between the distant nodes, the more critical these links become for the network. As expected, the criticality score of inter-community links was higher in the long-range flow scenario compared to the short-range flow scenario.

## Discussion

Percolation analysis is a powerful tool for understanding the global flow properties of networks. However, most conventional percolation-based analyses become less effective in the presence of a heterogeneous flow demand between different node pairs over the network. We have developed a method that makes use of a newly introduced percolation-driven property, namely, UD, in order to quantify network reliability. Based on the concept of UD, we presented a bottleneck identification scheme, that proved more effective than other state-of-the-art methods reported in the literature, in terms of both improving the reliability and reducing the delay imposed on flows by congested links. Note that the direct effect of congestion organization on travel time delay cannot be studied using the existing percolation models, because the removal of a congested link simply cannot help quantify the effect of its congestion on flow travel times. But it is an intriguing problem that suggests an important direction for future research.

Our proposed ideas are generally applicable to demand-serving networks including most physical infrastructures where there is an inherent demand for movement of an uneven amount of flow between different pairs of nodes in the network. With the ever-increasing availability of detailed data from real-world critical infrastructure networks, this study can be a helpful starting point for new research avenues and the development of more sophisticated theoretical tools to analyze flow demand, in order to achieve a more profound understanding of these complex systems.

## Methods

### Smart-card data

The data used in the real-world case study, are the smart-card transaction records, collected by the automated fare collection system for PT in Melbourne and Brisbane, Australia. Passengers are supposed to perform a scan-on transaction at the start and a scan-off at the end of their trip. Every smart-card transaction record contains multiple attributes, namely, anonymized card identifier, PT mode (bus, tram, or train), vehicle identifier (a unique number for each bus or tram vehicle), stop identifier, time-stamp, and transaction type (scan-on/off). For Melbourne’s network, we used an average of over 2,120,000 and 912,000 daily transactions associated with all PT modes on weekdays and weekends, respectively, collected during 61 days of September and October 2017. Brisbane data was collected during March 2013. After applying a cleaning process, we used the data to generate the temporal network of on-road PT supply and its corresponding passenger travel flow demand (see Supplementary Note 1 for details).

### Network and demand matrix construction

To generate the network representation of the on-road PT system on a particular day at time *t*, the structure and link attributes were estimated from the smart-card transactions time-stamped within the window [*t* − *δ*/2, *t* + *δ*/2]. The time window length *δ*, was set to 2 hours for experiments presented in the main article. First, we clustered the closely located PT stops and mapped each cluster to a node. Using information of smart-card transactions we derived the trajectory of every vehicle on the network, and if there was at least one vehicle traveling from one of the stops associated with node *i* to a stop associated with node *j* without stopping, we added a direct link *e*_*ij*_ starting at node *i* and pointing at node *j*. For each link *e*_*ij*_ the average travel time *τ*_*ij*_ over the time window was also calculated based on the information from the tracked vehicles. For a network of time *t*, demand matrix *F* measures the flow demand volumes by the number of O–D trips between nodes, within the time window used for the construction of the network. An O–D trip is a chain of one or more trip legs with transfers (but no activities) in between them. See Supplementary Note 1 on how single trip legs are chained to obtain O–D trips.

### Unaffected demand

To formulate the UD calculation, we use the so-called reachability matrix *R* = [*r*_*od*_] (the transitive closure of the network adjacency matrix) which is a square matrix of order *n*. Each entry *r*_*od*_ is equal to 1 if there is at least one directed path from node *o* to node *d* on the network, and *r*_*od*_ = 0 otherwise. Let *R*_*ρ*_ be the reachability matrix of network *G*_*ρ*_. At any threshold *ρ*, the amount of flow from *o* to *d* (*f*_*od*_) is deemed to be “unaffected” by link qualities *q* below the threshold *ρ* (*q* ≤ *ρ*) if there is at least one directed path from *o* to *d* remaining on *G*_*ρ*_, i.e., ${r}_{od}^{\rho }=1$. So, UD_*ρ*_ (defined as the unaffected proportion of the demand at threshold *ρ*) will be the sum of ${r}_{od}^{\rho }.{f}_{od}$ for all (*o*,*d*) pairs of nodes, normalized by the total flow demand

6 $${\rm{UD}}_{\rho }=\frac{{{\mathbf{1}}}_{n}^{\text{T}}({R}_{\rho }\circ F){{\mathbf{1}}}_{n}}{{{\mathbf{1}}}_{n}^{\text{T}}F{{\mathbf{1}}}_{n}}=\frac{{\text{tr}}(R_{\rho }F^{\text{T}})}{{{\mathbf{1}}}_{n}^{\text{T}}F{{\mathbf{1}}}_{n}},$$

where $\circ$ is the entry-wise product of matrices, tr(.) is the trace of the *n* × *n* square matrix, and **1**_*n*_ is a column vector of all *n* elements equal to one.

### The relation between the evolution of UD and GC during the percolation

Let ∣GC_*ρ*_∣ be the size of the GC as a function of *ρ*, then ∣GC_*ρ*_∣/*n* is called the incipient order parameter which is sometimes used to describe the connectivity of a fragmented network. If we assume a uniform flow demand distribution then on any undirected network, UD_*ρ*_ equals the proportion of connected node pairs in *G*_*ρ*_, which approaches ${(| {\rm{GC}}_{\rho }| /n)}^{2}$ as *n* → *∞*^43^. So, for large enough networks, monitoring the GC during the percolation is a special case of monitoring UD when flow demand is uniform. Therefore, we can accurately predict the evolution of ∣GC∣ during the percolation by assuming a uniform flow demand over the network and using $| {\rm{GC}}_{\rho }| \approx n.\sqrt{{\rm{UD}}_{\rho }}$. This is confirmed numerically in Fig. 7 and Supplementary Fig. 12.

Considering the above relation, when the demand is homogeneous (or unknown but assumed to be homogeneous), instead of the definition in Eq. (2) one may choose to use the area under the curve of ${\rm{UD}}_{\rho }^{1/2}$ as a reliability indicator that reflects the rate at which size of the connected components decline over the percolation process. However, our original definition in Eq. (2) has a simpler interpretation and it is mathematically tractable, allowing for theoretical analysis of network links in the simplest possible way.

### Link criticality score and its relation to network reliability

Suppose there exists a non-empty set of different directed paths Ψ_*od*_ that route between an origin node *o* and a reachable destination node *d*. During the percolation process on the network (whereby *ρ* is increased from zero to unity), each pathway *ψ* ∈ Ψ_*od*_ breaks up when the threshold *ρ* reaches to the minimum link-quality on that path. The “limiting link” associated with the flow from *o* to *d* (${e}_{od}^{* }$), when removed during the percolation process at $\rho ={q}_{od}^{* }$, breaks the last path(s) connecting *o* to *d* and affects the flow between them (*f*_*od*_). Using the definition of link criticality score in Eq. (4), we can expand the left-hand-side of Eq. (5) as

7 $$\sum _{{e}_{ij}\in E}{s}_{ij}.{q}_{ij}=\sum _{{e}_{ij}\in E}\sum_{o,d\in V,\atop {e}_{od}^{* }={e}_{ij}}\frac{{f}_{od}}{{{\mathbf{1}}}_{n}^{\text{T}}F{{\mathbf{1}}}_{n}}.{q}_{ij},$$

and for any pair *o*, *d* ∈ *V* with non-zero *f*_*od*_ there exist a single limiting link ${e}_{od}^{* }\in E$ with quality ${q}_{od}^{* }$, so

8 $$=\frac{1}{{{\mathbf{1}}}_{n}^{\text{T}}F{{\mathbf{1}}}_{n}}\sum _{o,d\in V}{f}_{od}.{q}_{od}^{* }.$$

During the percolation process, each entry in the reachability matrix *R*_*ρ*_ switches from 1 to 0 as soon as the last path(s) between its corresponding O-D nodes break. So, we can write

9 $${r}_{od}^{\rho }=\left\{\begin{array}{ll}1,&\rho\, <\, {q}_{od}^{* }\\ 0,&\rho \ge {q}_{od}^{* }\end{array}\right.,$$

where ${r}_{od}^{\rho }$ is the (*o*,*d*) entry of the reachability matrix *R*_*ρ*_ associated with the network *G*_*ρ*_. Note that the integral of ${r}_{od}^{\rho }$ with respect to *ρ* between the limits *ρ* = 0 and *ρ* = 1 is equal to ${q}_{od}^{* }$. So, from Eqs. (8) and (9) we can write

10 $$\sum _{{e}_{ij}\in E}{s}_{ij}.{q}_{ij}=\frac{1}{{{\mathbf{1}}}_{n}^{\text{T}}F{{\mathbf{1}}}_{n}}\sum _{o,d\in V}{f}_{od}.\int_{0}^{1}{r}_{od}^{\rho }d\rho ,$$

where the right-hand-side can be simplified with matrix operations to obtain Eq. (2) which is the definition of the reliability index *α*, so we can conclude that Eq. (5) holds. In Supplementary Note 3, the definition of the criticality score and the proof of Eq. (5) are generalized further, requiring no assumption on the link quality values.

## Supplementary information

Supplementary Information

Peer Review File

## Data Availability

Two weeks of Melbourne’s public transportation network data used in this study, are available at https://gitlab.com/homayoun/demand-serving-networks. Raw passenger smart-card data from Melbourne’s public transportation network were made available for research purposes by the associated transportation authority, which retains ownership over the data.

## Code availability

Source codes for the algorithms proposed in this study are available at https://gitlab.com/homayoun/demand-serving-networks. Specific codes that produce the results presented in this paper are available upon request.
