# Supplementary material for: Percolation of heterogeneous flows uncovers the bottlenecks of infrastructure networks
Source: Nat Commun. 2021 Feb 23;12:1254. doi: 10.1038/s41467-021-21483-y (PMC7902621; doi:10.1038/s41467-021-21483-y)
Supplement: Supplementary file 1 — Supplementary Information [file 41467_2021_21483_MOESM1_ESM.pdf]

Supplementary Information for

Percolation of heterogeneous flows uncovers the bottlenecks of infrastructure networks

Homayoun Hamedmoghadam*, Mahdi Jalili, Hai L. Vu and Lewi Stone*

Homayoun Hamedmoghadam. E-mail: homayoun.hamed@monash.edu
Lewi Stone. E-mail:lewistone100@gmail.com

The SI includes:

Supplementary Notes 1 to 7
Supplementary Figures 1 to 12
Supplementary References

Supplementary Note 1: Smart-Card Data Processing

Tracking vehicles. We tracked bus and tram vehicles based on temporally sorted sequence of stop ID-timestamp pairs associated with smart-card transactions made on each vehicle. Each vehicle trajectory corresponds to a sequence of visited stops with arrival, departure, and dwelling time at each stop, which are respectively the timestamp for first transaction, last transaction, and the time span between the first and last transactions within the uninterrupted sequence of transactions made on the vehicle at a particular stop. If there was only one transaction recorded for a vehicle's visit to a stop, the dwelling time was considered to be from 10 seconds before to 10 seconds after the timestamp of that transaction.

Spatial clustering of stops. To construct the network representation of the Public Transportation (PT) system, we first performed a simple spatial clustering on the stops, and then mapped each stop cluster to a node on the network. This allowed us to deal with the existence of closely located stops with no direct transit service connecting them. This can be the case at intersections, PT hubs where different routes meet, and different sides of a street that often serve the same route but in different directions. The goal is to make stop clusters consisting of the stops so close to each other that no route would be designed to serve more than one of them and no passenger would use any transportation mode other than walking, to move from one to the other. To do so, we considered a convenient walking distance threshold of 200 m and in a recursive process we merged close stops together so to include every pair of stops with a distance of 200 m or less into the same cluster. As there is a chance that a chain of spatially close stops leads to a large cluster, we limited the clusters to have a maximum of 100 m radius; i.e. the maximal distance of the cluster members from the cluster centroid. Then, for clusters of radii larger than 100 m, we repeatedly removed the point with the largest distance from the centroid until the radius satisfies the condition. Then, we applied the clustering process to the points removed from the clusters in the previous step, and repeated the whole procedure until all points are assigned to clusters.

Network construction. To generate the network representation of the PT system, we first mapped each stop cluster to a node. Then, if there was at least one vehicle traveling from node i to node j, we added a directed link e_{ij} from node i and pointing to node j. Each link represents a service in the PT system between two immediate nodes. For any particular time t during the day, the average travel time attribute τ_{ij} of the link e_{ij} can be calculated based on the data from the vehicles travelling on the link during the interval $[t-\delta/2, t+\delta/2]$ where δ is length of the time window, discussed further in the followings. Travel time attribute of links was derived from vehicle trajectories as the average elapsed time for vehicles since departure from the source node until arrival time at the target node.

Trip chaining. An Origin-Destination (O-D) trip is a journey between two anchor points consisting of a single trip leg, or multiple trip legs entailing transfers in-between. The process of connecting trip legs at transfer points to estimate O-D trips (also called *journeys*) is called *trip chaining.* The O-D flow demand in transportation networks, often called O-D travel demand or O-D passenger flow demand, is explained by the number of O-D trips between each O-D pair of nodes in the network over a certain time window. Trip chaining for PT passenger trips is usually performed based on some assumptions about the behavior of PT users. The only assumption we make here is that PT users do not use any mode of transportation other than walking between two consecutive trip legs, i.e. at the interchange point. Thus, transfers should include nothing more than a walk from the alighting stop to the next boarding stop and waiting for the connection. The problem is to find an optimal *allowable transfer time* to determine whether a transfer or an activity is carried out by the passenger between consecutive trip legs (from alighting time to the next boarding time).

We developed an unsupervised learning approach to classify the times spent by passengers at interchange points (inter-transaction times) into transfers and activities. Let us define a return trip as a sequence of trip legs which starts with boarding at a reference point and ends with alighting at a close proximity of the reference point, while the maximum inter-transaction time corresponds to the interchange point with the largest geodesic distance from the reference point. The idea is that each return trip includes undertaking an activity at that particular target point. We first detected the return trips separately

on the first five weekdays and the first five weekend days in September 2017. Then, we built the histogram of transfer and activity duration from the inter-transaction times extracted from the return trips, separately for weekdays (Supplementary Figure 1A) and weekends (Supplementary Figure 1C). The histogram shows a large number of transfers associated with small inter-transaction times, while the number of observed transfers decreases drastically with increasing inter-transaction duration. The activity duration histogram for weekdays depicts three local maxima, approximately at 1.5, 7, and 8.5 hours; see Supplementary Figure 1A. The first peak is associated with common daily non-occupational activities. The two other peaks roughly at 7 and 8.5 hours long, are in surprising agreement with regular school day and daily work duration that are 6.5 hours (1) and 7.6 hours (2), respectively. The peak associated with school disappears in weekend inter-transaction time distribution while a small local maximum remains at the duration associated with work.

Next, we find the allowable transfer time θ, to label inter-transaction times equal or less than the threshold as transfers, and the rest as activities. Labeling the inter-transaction durations can be viewed as a dichotomous (binary) classification problem where the positive samples, i.e. samples that should have been labeled as positive, are the transfers and activities are the negative samples. In order to choose the optimum threshold θ to perform the classification, we used the Youden's J statistic (also called *Informedness*) (3), which measures the accuracy of a dichotomous (binary) classifier and is defined as below:

$$Informedness = Recall + Inverse\ Recall - 1, \tag{1}$$

where $Informedness \in [0, 1]$ is the probability of an informed decision as opposed to a random guess taking into account all predictions made by the classifier. Assuming that the actual labels for transfers are positive and activities are negative, *Recall* and *Inverse Recall* can be calculated as:

$$Recall = P(W \leq \theta), \tag{2}$$

$$Inverse\ Recall = P(V > \theta), \tag{3}$$

where W and V are the random variable corresponding to derived transfer and activity durations, respectively.

The optimal θ maximizing the *Informedness* was found to be 43 minutes for weekdays (Supplementary Figure 1B) and surprisingly the exact same threshold was found for weekends (Supplementary Figure 1D). Therefore, to build O-D trips for each passenger, first the trip legs associated with a particular card ID were sorted chronologically. Then, each pair of consecutive trips belong to a single O-D trip if the inter-transaction time between the subsequent legs did not exceed the determined inter-transaction time threshold $\theta = 43$ min, implying a transfer at the interchange point. There was an average of more than 33,000 transfers per working day and about 10,000 daily transfers during weekends. Finally, an O-D trip was described with two location-timestamp pairs associated with the boarding of the first leg (origin) and alighting of the last leg (destination) for a chain of trip legs.

Estimating missing alighting transactions. The information of a passenger trip is complete only when there is full information from a boarding scan-on record and an alighting scan-off record. Unpaired transactions are a common problem for AFC data, due to the nature of AFC systems as they depend on human actions which involve errors, and also their various components, e.g. reading and recording, which can malfunction. However, missing transactions can be estimated with high accuracy using the information of immediate previous and following transactions (4, 5).

We deployed a procedure to estimate the missing alighting transactions, where for a particular card ID, there is a scan-on transaction on a bus or tram without a valid paired scan-off later on the same vehicle (missing alighting), but the next transaction recorded for that card is a scan-on (second boarding) made that day or the next day on any PT mode (see Supplementary Figure 1E). By following the boarded vehicle's trajectory starting from the first boarding timestamp until the second boarding timestamp, we generated a set of candidate alighting points by filtering the stops visited by that vehicle. Candidate alighting stops are within 2 km radius of the second boarding point, and it is possible to walk from them to the second boarding point with the speed of 4.5 km/h within the inter-transaction duration. An allowable transfer time is already calculated, which classifies the purpose of the alighting-then-boarding events into transfers and activities. If alighting at the candidate stop with the shortest Euclidean distance to the second boarding stop, allows undertaking an activity, it was identified as the missing alighting point. Otherwise, the candidate stop which allows the earliest arrival of the passenger to the second boarding stop was identified as the missing alighting point.

Choice of time window length and O-D flow demand generation. Here, we discuss the effect of time-window length on the generated network and the O-D travel flow demand matrix F. For each time window within the day, the travel time of a link was calculated by aggregating the travel time information of multiple vehicles traversing the link. The aggregation time window should be long enough, so the link qualities fairly represent the impact of phenomena, such as signals, which might affect each vehicle differently. Furthermore, if the window is too small, the network links associated with low frequency services will be intermittent on the temporal network structure. However, during a long interval, conditions such as a transient traffic congestion might change and the link qualities will not reflect the temporary conditions accurately if the time window is too wide. To count the number of trips between O-D pair of nodes on the network, the time window should be large enough to encompass the trips on the network.

A 2-hour time window was found to be large enough to observe at least one vehicle on the links with low service frequency, while encompassing almost all O-D trips on the network (see Supplementary Figure 1F). Additionally, it is not too large to conceal or smooth out the transient road conditions in resulting link qualities. As such, we chose the aggregation time window length of 2 hours for the experiments presented in *Main Text* and also here, unless otherwise stated. It is worth mentioning that

a 2-hour time window as maximum duration of most trips, is also recognized by Melbourne's PT authority (Public Transport Victoria), and passengers do not have to pay additional fares for 2 hours after each payment made on a scan-on transaction.

After generating the network representation of the on-road PT system at time t on a particular day based on PT services running within the time interval $[t-\delta/2, t+\delta/2]$, we followed the next steps, namely, determining the maximum allowable transfer time, estimating the missing alighting information, and applying trip chaining process, to obtain O-D trips during the same time interval. The $n \times n$ matrix F for network of size n, was generated, where each entry (o, d) of the matrix, denoted as f_{od}, is the number of O-D trips (chained trips or passenger journeys) form node o to node d within the target time window.

Supplementary Note 2: Involving flow-capacity of links in network reliability analysis

We proposed a new approach to monitor the percolation process, which provides a quantitative insight on network reliability, in terms of the ability to provide paths of high-quality links for its flows with respect to the demand distribution. Our network analysis in the *Main Text* is not concerned with capacity of the network. The reason is that our analysis pinpoints the links with problematic congestion levels, and any capacity-related problem can be seen independent of that and may be solved completely in parallel. To make this clear with an example related to PT networks, let us imagine that the capacity of some PT vehicles is increased. This increases the maximum flow-capacity of the network, yet it does not result in any change to the demand and link qualities over the network, thus, the result of our reliability analysis will remain unchanged. So, one can use our analysis to study the network congestion in relation to demand distribution and pinpoint the problems with the network, but then the any problem related to capacity of links can be attended completely in parallel or independently. Nevertheless, here we demonstrate that the proposed framework can be extended to involve the flow-capacity of network links in reliability analysis. In particular, definition of the Unaffected Demand (UD) can be extended to study the ability of networks to provide high-quality paths and "accommodate" the demand on such paths.

The amount of demand on the network under percolation. Recall that at any threshold ρ during the percolation process, our proposed $UD(\rho)$ is the proportion of the network's flow-demand between the Origin-Destination (O-D) node pairs that remain connected only by links of quality above the threshold ρ ($q_{ij} > \rho$). At each threshold ρ during the percolation process, subnetwork G_ρ is generated by inheriting all the links with quality q above the threshold ($q > \rho$) from the network G. We defined $R_\rho = [r^\rho_{od}]$ as the reachability matrix of subnetwork G_ρ (see *Methods* in the *Main Text*). Using the reachability matrix, for the network G with total demand of $\sum_{o,d \in E} f_{od}$ the amount of remaining demand on its subnetwork G_ρ is $\sum_{o,d \in E} r^\rho_{od}.f_{od}$, where f_{od} is the volume of demand from node o to node d; note that $UD(\rho) = (\sum_{o,d \in E} r^\rho_{od}.f_{od}) / \sum_{o,d \in E} f_{od}$.

Calculating the flow-capacity of the network under percolation. From the data, we derived the number of buses/trams running on each link at each snapshot of the network in time. We assumed the capacity of each on-road PT vehicle to be 50 passengers which is a conservative choice (a normal sized bus can practically take 70-80 passengers). Then, at each threshold ρ in the percolation process we approximated the capacity of the subnetwork G_ρ as explained in the following. Take a very small λ (close to zero) so that the network G_ρ has the capacity for concurrent movement of $\lambda.r^\rho_{od}.f_{od}$ passengers between all (o, d) node-pairs (r^ρ_{od} is zero if o and d are disconnected). The variable λ can be increased until it reaches a maximum before it becomes impossible for network links to match the amount of flows. We denote the maximum possible value of λ for the network G_ρ by $\lambda_{max}(\rho)$. The problem of finding λ_{max} is known as "maximum concurrent multicommodity flow" problem, which is strongly NP-complete but can be approximated in polynomial time by a number of algorithms. Here, we used a modified version of Fleischer's algorithm (6) which is fast and accurate enough for our purpose, and provides a lower approximation of the maximum flow-capacity of the network. For any network with given link capacities and O-D flow demand, Fleischer's algorithm chooses a priority path between each O-D pair, assigns a small proportion of the demand between the O-D pair to all the links on that path, and updates the capacity of those links. The algorithm iteratively augments flows to the network links (which corresponds to increasing λ), and terminates when augmentation becomes impossible and returns the final λ as λ_{max}. At any threshold ρ if the algorithm returns, say, $\lambda_{max}(\rho) = 0.1$, it means that G_ρ can accommodate the concurrent flow of 10% of the demand between each O-D pair, but if $\lambda_{max}(\rho) \geq 1$ then G_ρ is capable of accommodating the whole demand between its connected O-D pairs. We define $C(\rho) = \lambda_{max}(\rho).UD(\rho)$ to simply compare the capacity $C(\rho)$ and the demand $UD(\rho)$ on G_ρ, both normalized by the total amount of demand on network G_0.

We checked the change in capacity of the real on-road PT networks during the percolation process and observed that as congested links are progressively being removed, the capacity of the network never falls below the amount of demand corresponding to UD. This is not surprising, as although some routes can become very crowded at peak hours, on average the utilization of on-road PT vehicles are generally low even in large cities (vehicles are not operating close to their capacity). As an example, in Supplementary Figure 2 the black curve shows the evolution of $UD(\rho)$ during the percolation process on a snapshot of the Melbourne's network at rush-hour (the same curve as in Fig. 2d of the *Main Text*), and the capacity $C(\rho)$ depicted via the red curve demonstrating that the network can handle 1.5 to 3 times of the $UD(\rho)$ at any point in the percolation process. The network during non-rush hours has even a higher capacity relative to its demand, as the drop in the number of PT services is less than the decline of total passenger flow-demand from rush to non-rush hours.

Involving the capacity into percolation analysis. If a demand-serving network functions close to its flow-capacity and one wants to study the problems with capacity of the network in addition to the conflict between flows and congestion, then, our definition of UD can be extended to $UD_c(\rho) = \min\{UD(\rho), C(\rho)\}$. The new capacity-aware unaffected demand (UD_c), monitors the

proportion of the total demand that can be "accommodated" between O-D pairs only on links with quality above the threshold ρ; during the percolation always $UD_c(\rho) \leq UD(\rho)$, and $UD_c(\rho) < UD(\rho)$ if O-D paths on G_ρ cannot match the remaining demand over the subnetwork. Accordingly, the reliability measure α can be extended to capacity-aware reliability α_c defined as the area under the curve of $UD_c(\rho)$ over $\rho \in [0, 1]$, i.e. $\alpha_c = \int_0^1 UD_c(\rho)\, d\rho$.

Supplementary Note 3: Link Quality, Link Criticality Score, and Network Reliability

In this note we first provide the general definition of the link criticality score, given that it is possible for links to have equal quality attributes. Then, we prove that the relationship between link quality q, link criticality score s, and the proposed percolation-based demand-serving reliability of networks α, given by Eq. 5 in the *Main Text*, still holds. We end this note by giving analytical proof to the effectiveness of using link criticality score for the problem of network bottleneck identification. In the following we try to provide sufficient examples to assure the comprehensibility and reproducibility of the proposed method.

Link criticality score. The definition of link criticality score in Eq. 4 of the *Main Text* assumes unique link quality values over the network. To be generally applicable though, it needs to be modified to take into account the possibilities that i) for a single O-D pair there are multiple connecting pathways each include a different link with quality equal to q^*_{od}, and ii) there are multiple minimum-quality links on a single connecting pathway.

Let ψ be a sequence of links corresponding to a directed path on the network. For any path ψ, we define the *path quality* (q_ψ) as the minimum quality among all links on that path; i.e. $q_\psi = \min_{e_{ij} \in \psi} q_{ij}$. We deem a network as reliable, where despite presence of local (link-level) perturbations, reflected as lowered link qualities, the network provides alternative paths with high-quality links for the flows between O-D nodes. Therefore, for an origin node o and a reachable destination node d on the network $(o, d \in V)$, from a non-empty set of all directed paths connecting them, denoted by Ψ_{od}, we take the maximum quality path(s) as the primary (optimal) path(s) for flows between (o, d) pair. The optimal path maximizes the minimum link-quality on the pathways from o to d. Let us define the set of all optimal paths between a pair of O-D nodes as:

$$\sigma_{od} = \underset{\psi \in \Psi_{od}}{\operatorname{argmax}} \; q_\psi. \tag{4}$$

The minimum link-quality on all paths (path quality) in σ_{od} are equal to q^*_{od}. So, paths in σ_{od} break at threshold $\rho = q^*_{od}$ and make node d unreachable from node o; i.e. the flow volume f_{od} becomes affected. As an example, in Fig. 3a-c of the *Main Text*, $\sigma_{1,4}$ includes $1 \to 2 \to 3 \to 4$ and $1 \to 5 \to 2 \to 3 \to 4$ with $q^*_{1,4} = q_{2,3} = 0.6$.

Considering the quality of links as their weights, finding the set $\sigma_{od} \subseteq \Psi_{od}$ on the weighted network is known as the maximum capacity paths problem (7). The optimal path(s) between each node pair can be found via a modified version of a conventional shortest path algorithm; e.g., Dijkstra's algorithm (8) should be modified to compare paths based on their minimum link-quality and then prefer the path with the largest value of the minimum link-weight. With optimal paths between all O-D pairs of nodes found, the generalized definition of criticality score s_{ij} for the link e_{ij}, free from any assumption, can be formulated as:

$$s_{ij} = \sum_{o,d \in V} \sum_{\psi \in \sigma_{od}} \frac{f_{od}.\lambda(e_{ij}, \psi)}{(\mathbf{1}_n^{\mathrm{T}} F \mathbf{1}_n).|\sigma_{od}|.|\epsilon_\psi|}, \tag{5}$$

where $\lambda(e_{ij}, \psi)$ is equal to 1 if e_{ij} is (one of) the minimum-quality link(s) on the path ψ, and 0 otherwise. And $\epsilon_\psi = \{e_{kl} \in \psi | q_{kl} = q_\psi\}$ is the set of all links e_{kl} on the path ψ, that have equally the minimum link-quality on the path ψ. In practice, all (o, d) pair of nodes can be visited to first find q^*_{od} according to all paths in Ψ_{od}, and then the set of optimal directed paths $\sigma_{od} \subseteq \Psi_{od}$ connecting each pair, and finally the set of minimum-quality link(s) ϵ_ψ on each path $\psi \in \sigma_{od}$, to allow calculation of all criticality scores s_{ij} using Supplementary Equation 5. If a link e_{ij} is not found as the minimum-quality link on the optimal path between any (o, d) pair with $f_{od} > 0$, or in other words if it never found as the limiting link associated with a (o, d) pair with $f_{od} > 0$, then s_{ij} will be 0.

The proportion of the total demand flowing from node o to node d is $f_{od}/(\mathbf{1}_n^{\mathrm{T}} F \mathbf{1}_n)$, which indicates the importance of the connectivity between them. If there is a single optimal path connecting o to d with a single minimum-quality link on the path, the proportion of the total flow demand between the pair (o, d) is fully added to the criticality score of that link. For the example of Fig. 3 in the *Main Text*, $s_{2,3}$ is the proportion of the total demand that is between six node pairs, as shown in Supplementary Figure 3*B*. In case there are multiple optimal paths from node o to d, i.e. $|\sigma_{od}| > 1$, the flow demand proportion $f_{od}/(\mathbf{1}_n^{\mathrm{T}} F \mathbf{1}_n)$ is divided equally between those paths and share of each path is added to the criticality score of the limiting link on that path. For the network seen in Fig. 3 (*Main Text*), $|\sigma_{1,4}| = 2$ but the single limiting link $e_{2,3}$ for the node pair $(o, d) = (1, 4)$ is on both optimal paths, thus ultimately $f_{od}/(\mathbf{1}_n^{\mathrm{T}} F \mathbf{1}_n)$ is fully contributed to $s_{2,3}$. If there are multiple minimum-quality links on an optimal path $\psi \in \sigma_{od}$, i.e. $|\epsilon_\psi| > 1$, the share of each optimal path, i.e. $f_{od}/((\mathbf{1}_n^{\mathrm{T}} F \mathbf{1}_n).|\sigma_{od}|)$, is divided equally between those equally-minimum-quality links.

Deriving the key identity for α. Here, we show that the relationship between link qualities, link criticality scores, and the demand-serving reliability of a network, expressed by Eq. 5 of the *Main Text*, also holds for the general definition of the criticality score in Supplementary Equation 5. The significance of this identity is that it allows us to derive the impact of

increasing the quality of links (improving links) on network reliability. Using Supplementary Equation 5 we can write:

$$\sum_{e_{ij}\in E} s_{ij}.q_{ij} = \frac{1}{\mathbf{1}_n^{\mathrm{T}}F\mathbf{1}_n}\sum_{o,d\in V}\frac{f_{od}}{|\sigma_{od}|}\sum_{\psi\in\sigma_{od}}\sum_{e_{ij}\in E}\frac{q_{ij}.\lambda(e_{ij},\psi)}{|\epsilon_\psi|} \tag{6}$$

and as for each path ψ, when iterating over all links in the network, $\lambda(e_{ij},\psi)$ becomes 1 for only links from the set of minimum-quality links on ψ,

$$= \frac{1}{\mathbf{1}_n^{\mathrm{T}}F\mathbf{1}_n}\sum_{o,d\in V}\frac{f_{od}}{|\sigma_{od}|}\sum_{\psi\in\sigma_{od}}\sum_{e_{ij}\in\epsilon_\psi}\frac{q_{ij}}{|\epsilon_\psi|} \tag{7}$$

$$= \frac{1}{\mathbf{1}_n^{\mathrm{T}}F\mathbf{1}_n}\sum_{o,d\in V}\frac{f_{od}}{|\sigma_{od}|}\sum_{\psi\in\sigma_{od}}\sum_{e_{ij}\in\epsilon_\psi}\frac{\min\limits_{e_{kl}\in\psi} q_{kl}}{|\epsilon_\psi|} \tag{8}$$

and as $|\epsilon_\psi|$ is the number of links which are equally the minimum-quality links on the path ψ,

$$= \frac{1}{\mathbf{1}_n^{\mathrm{T}}F\mathbf{1}_n}\sum_{o,d\in V}\frac{f_{od}}{|\sigma_{od}|}\sum_{\psi\in\sigma_{od}}\min_{e_{kl}\in\psi} q_{kl} \tag{9}$$

and for any optimal directed path ψ connecting o to d ($\psi\in\sigma_{od}$), the minimum link-quality is equal to q^*_{od}, so

$$= \frac{1}{\mathbf{1}_n^{\mathrm{T}}F\mathbf{1}_n}\sum_{o,d\in V} f_{od}.q^*_{od} \tag{10}$$

Supplementary Equation 10 is identical to Eq. 8 of the *Main Text* which is then manipulated in a couple of steps to conclude (see *Methods* section of the *Main Text*):

$$\sum_{e_{ij}\in E} s_{ij}.q_{ij} = \alpha \tag{11}$$

Let us validate the above equation in an example. For the toy network of Fig. 1 (*Main Text*), paths from node 1 to node 2 are $1\to2$ and $1\to4\to2$. Links with the minimum quality on these paths are $e_{1,2}$ and $e_{1,4}$, among which $e_{1,4}$ has the maximum quality; thus $1\to4\to2$ is the optimal path. Having $f_{1,2}=5$ and the total flow demand of 100, contributes 0.05 to $s_{1,4}$. The two paths $2\to3\to5$ and $2\to1\to3\to5$ connect nodes 2 and 5, and their minimum link-qualities are respectively $q_{2,1}=q_{3,5}=0.6$ and $q_{2,3}=0.3$. Therefore, the former is the optimal path, but as it has two equally minimum-quality links, the flow demand proportion $f_{2,5}/100=0.03$ is divided by two and then 0.015 is added to both $s_{2,1}$ and $s_{3,5}$. Link $e_{1,2}$ is not the minimum-quality link of any optimal path thus $s_{1,2}=0$. Link $e_{1,3}$ is the minimum-quality link on the optimal path connecting node 1 to node 3, and also together with $e_{5,1}$, they are the minimum-quality links on the optimal path connecting node 5 to 3, thus $s_{1,3}=(f_{1,3}+f_{5,3}/2)/100=0.105$. Other non-zero link criticality scores on the network are $s_{1,4}=0.25$, $s_{2,1}=0.14$, $s_{2,4}=0.11$, $s_{3,5}=0.21$, $s_{4,2}=0.1$, and $s_{5,1}=0.085$. Therefore, the sum of $s_{ij}.q_{ij}$ over all links (left-hand side of Supplementary Equation 11) equals 0.65, which is equal to the area under curve of UD_ρ which equals α (right-hand side of Supplementary Equation 11) as expressed by Eq. 2 of the *Main Text*; see also Fig. 1b of the *Main Text* for the area under curve of UD_ρ for this example network. Supplementary Figure 3*B* visualizes the relationship between link qualities q, link criticality scores s, and the demand-serving reliability α using this example network.

Implication of link criticality score in reliability of the network. Consider increasing the quality q_{ij} of a chosen link e_{ij} to q'_{ij} ($q'_{ij}>q_{ij}$). Here, we give the full details to prove that there exists a non-empty range of values for q'_{ij}, for which criticality score of the whole network will remain unchanged. Thus, according to Supplementary Equation 11, increasing the quality of the link e_{ij} from q_{ij} to q'_{ij} ($\Delta q_{ij}=q'_{ij}-q_{ij}$) within the abovementioned range, increases the reliability of the network, directly proportional to the criticality score of the link s_{ij}, by exactly $s_{ij}.(q'_{ij}-q_{ij})=s.\Delta q_{ij}$.

Let us assume again that for two different links $e_{ij}\neq e_{kl}$ on the network, the probability of having equal qualities is zero, i.e. $P(q_{ij}=q_{kl})=0$. This in theory is correct assuming that link qualities come from a continuous distribution, and is approximately correct in practice when link qualities are calculated as decimals with high precision. Based on this assumption, link criticality scores can be calculated as Eq. 4 (in the *Main Text*), which simply expresses that s_{ij} is the proportion of total flow demand between the O-D pairs for which e_{ij} is the limiting link. Let us denote the set of all ordered pairs of network nodes (o,d), where $o,d\in V$ and $o\neq d$, with non-zero flow demand ($f_{od}>0$), as V_p. Our approach is to investigate the impact of increasing the quality q_{ij} of link e_{ij}, on three mutually disjoint subsets of V_p, denoted as V^p_c, $c=1,2,3$, with the property:

$$V^p = \bigcup_{c=1,2,3} V^p_c(e_{ij}), \quad \forall e_{ij}\in E. \tag{12}$$

From all (o,d) pairs in V^p:

- $V_1^p(e_{ij})$ includes those for which e_{ij} is the minimum-quality link on the optimal path(s) $\psi \in \sigma_{od}$ connecting o to d ($q_{ij} = q_\psi = q^*_{od}$),
- $V_2^p(e_{ij})$ include those for which e_{ij} does not appear on optimal paths, but it is part of at least one connecting path within which q_{ij} is the minimum link-quality,
- $V_3^p(e_{ij})$ includes those for which e_{ij} does not appear on any path connecting o to d, or if it does, q_{ij} is larger than the path quality (minimum link-quality on the path).

For any link e_{kl} same as or different from e_{ij}, criticality score s_{kl} can be calculated as:

$$s_{kl} = \sum_{c=1,2,3} \sum_{\{(o,d)\in V_c^p(e_{ij})|e^*_{od}=e_{kl}\}} \frac{f_{od}}{\mathbf{1}_n^{\mathrm{T}} F \mathbf{1}_n}. \tag{13}$$

Note that criticality score of any link can be written as the summation of three components associated with $c = 1, 2, 3$. For a network with initial demand-serving reliability of α, after increasing the quality of e_{ij} from q_{ij} to q'_{ij}, the new reliability will be α' ($\alpha' \geq \alpha$). Next, we show that under certain conditions for q'_{ij} all link criticality scores will remain unchanged, which allows the exact calculation of α'.

Increasing q_{ij} does not change the limiting link of any (o, d) pairs in $V_3^p(e_{ij})$, hence for all links the component associated with $c = 3$ in Supplementary Equation 13 remains unchanged. Let $\hat{V}_2^p(e_{ij})$ be a subset of (o, d) pairs in $V_2^p(e_{ij})$, for which there is at least one path connecting o to d including e_{ij} where quality of all other links is above q^*_{od}, formally defined as:

$$\hat{V}_2^p(e_{ij}) = \{(o,d) \in V_2^p(e_{ij}) | \exists\psi \in \Psi_{od} : (e_{ij} \in \psi) \wedge (q_{kl} > q^*_{od})\}. \tag{14}$$

Then, as long as $\forall(o, d) \in \hat{V}_2^p : q'_{ij} < q^*_{od}$, e_{ij} (with its new quality q'_{ij}) cannot take over the role of limiting link between any O-D pair and the component associated with $c = 2$ of Supplementary Equation 13 for criticality score of all links remains unchanged. Any link e_{ij} with $s_{ij} = 0$, is not the limiting link between any node pair with non-zero flow demand, i.e. $V_1^p(e_{ij}) = \emptyset$, thus we can conclude that:

$$s_{ij} = 0 \wedge q'_{ij} < \min(\{q^*_{od} | (o,d) \in \hat{V}_2^p(e_{ij})\} \cup \{1\}) \Rightarrow \alpha' = \alpha. \tag{15}$$

Let q^{**}_{od} be the second lowest link-quality on all optimal paths connecting o to d. For a link e_{ij} with $s_{ij} > 0$, we have $V_1^p(e_{ij}) \neq \emptyset$, and the component associated with $c = 1$ in Supplementary Equation 13 will remain fixed as long as e_{ij} maintains its role as the limiting links between all pairs in $V_1^p(e_{ij})$, i.e. if $\forall(o, d) \in V_1^p(e_{ij}) : q'_{ij} < q^{**}_{od}$. Therefore, with the help of proved identity in Supplementary Equation 11 we can write:

$$s_{ij} > 0 \wedge q'_{ij} < \min(\{q^*_{od} | (o,d) \in \hat{V}_2^p(e_{ij})\} \cup \{q^{**}_{od} | (o,d) \in V_3^p(e_{ij})\}) \Rightarrow \alpha' - \alpha = s_{ij}.(q'_{ij} - q_{ij}). \tag{16}$$

To summarize all the above, for any link with criticality score of zero there exists a non-empty range of values for increased link criticality score q'_{ij} that network reliability will certainly remain unchanged $\alpha' = \alpha$. However, if the link criticality score is larger than zero, there exists a non-empty range of q'_{ij} values, for which no link criticality score changes in the network. Thus, according to the key relationship identified between q, s, α (Supplementary Equation 11 here or Eq. 5 in the *Main Text*), increasing q_{ij} to q'_{ij} within a certain non-empty range, changes the reliability of the network to $\alpha' - \alpha = s_{ij}.(q'_{ij} - q_{ij})$, that is directly proportional to the criticality score of the ameliorated link. Thus, one can generally expect further improvement on the network reliability from amelioration of the links with higher criticality scores. Accordingly, network bottlenecks can be identified as links with the highest criticality scores. For the network in Fig. 1 of the *Main Text*, the link with the highest criticality score is $e_{1,4}$ which if completely ameliorated ($q_{1,4} = 0.4$ and $q'_{1,4} = 1$), will improve the reliability of the network from $\alpha = 0.65$ to $\alpha' = 0.768$.

Supplementary Note 4: Implication of Heterogeneous O-D Flow Demand

A drawback of the percolation threshold ρ_c as a reliability index can be noticed through consideration of heterogeneous distribution of flow demand, where the volume of in-demand flow is not equal between all pairs of nodes. In presence of a heterogeneous flow demand, the pathways connecting O-D pairs with high demand, are of more importance than those with low or no flow demand. After percolation criticality (subcritical phase), when the Giant Component (GC) is fragmented into small and medium-sized clusters, heterogeneous flow demand might allow a significant portion of flows to still be preserved within isolated clusters. Or on the contrary, even before criticality (supercritical phase) a significant portion of the in-demand flows might be unable to reach their destination due to, for example, insignificant fragmentations of the GC leading to isolation of O-D nodes with very large demand volume.

Here, we investigate the implication of the heterogeneous demand, by demonstrating that during the percolation process on the Melbourne's on-road PT network, actual in-demand O-D trips do not break with the same rate as O-D pairs of nodes become disconnected. In the proposed framework, Unaffected Demand (UD) is defined as the proportion of total flow demand that can reach the destination node from the origin at any point during the percolation process; see *Main Text* for details. On any network, if the O-D flow demand is uniformly distributed over all reachable O-D pairs of nodes, then at any threshold ρ, UD will be equal to the proportion of reachable node pairs on the network. Having a uniform demand means that the total

flow demand is divided up equally between all reachable O-D pairs of nodes on the network. Let us denote the UD of the special case of having a uniform flow demand, as UD', which as a function of ρ can be calculated as below:

$$UD'_\rho = \frac{\mathbf{1}_n^\mathrm{T} R_\rho \mathbf{1}_n}{\mathbf{1}_n^\mathrm{T} R_0 \mathbf{1}_n} \tag{17}$$

Supplementary Equation 17 can be derived from Eq. 6 of the *Main Text*, when the flow demand matrix F has a constant value in all its entries associated with reachable O-D nodes on the network.

Generally, during the percolation, UD' decreases as O-D pairs of nodes, separated by lower quality links, become progressively disconnected. Given that , if $\gamma < 1$, then UD decreases faster than UD' during the percolation, meaning that the flow demand is relatively more between O-D node pairs separated by lower quality links. And if $\gamma > 1$, then there is relatively more demand to flow between O-D pairs connected by paths of higher quality links. Supplementary Figure 5 compares the evolution of UD_ρ (the proportion of not-yet-broken O-D trips) with UD'_ρ (the proportion of not-yet-separated node pairs) during the percolation on Melbourne's PT network, separately for weekdays (Supplementary Figure 5*A*) and weekends (Supplementary Figure 5*B*). The figure demonstrates that $\gamma > 1$ during both weekdays and weekends, meaning that there was a relatively high passenger travel flow demand between the O-D pairs connected through pathways made up of higher quality links. Furthermore, the observed phenomenon was magnified during weekdays ($\gamma = 4.64$) compared to weekends ($\gamma = 3.66$). This finding is consistent with higher demand-serving reliability α of weekdays compared to weekends, as it shows that the loss of connectivity during the percolation affected the demand with a slower rate in networks of weekdays compared to those of weekends.

Supplementary Note 5: Percolation on Melbourne's PT Network

We calculated the critical threshold ρ_c and demand-serving reliability α of the on-road PT network of Melbourne, during the daily active period of the system, i.e. 4:00-24:00, in steps of 30 min length. In Supplementary Figure 6, ρ_c (Supplementary Figure 6*A*&*B*) and α (Supplementary Figure 6*C*&*D*) are depicted as a function of the time t of the day. Supplementary Figure 6*A*&*C* show the evolution of the indices over each single weekday (curve color shows the date) during the two months of September and Octorber 2017. Supplementary Figure 6*B*&*D* plot ρ_c and α versus time t, averaged at each time-point over 43 (18) working days (days off) during the course of the available data. Also, the envelope of a single standard deviation of ρ_c and α values (calculated at each time of the day over two months) is shown by the shaded areas, below and above the mean. The standard deviation of α values is particularly small, making it possible to easily discern a general trend in the average α, as it changes over the day (the signal to noise ratio is high). Because of the small standard deviation, the same trend will observable in the evolution of α over any single day. This is corroborated by the plots of the raw timeseries over each single day in Supplementary Figure 6*C*. In contrast, in Supplementary Figure 6*B*, we see that the standard deviation of ρ_c is larger than fluctuations in the average trend for ρ_c (averaged at each time t over the two months), plotted over a day. Thus, the trends in the average signal of ρ_c, are dominated by the fluctuations of the noise (the signal to noise ratio is very low). The temporal evolution of ρ_c over a single day will not resemble the hourly averaged data seen in Supplementary Figure 6*B*. This is corroborated by the plots of the raw timeseries during each single day in Supplementary Figure 6*A*, where the effect is accentuated even more strongly. Thus, ρ_c exhibits large fluctuations in comparison to the smooth temporal evolution of α. In the real world under normal conditions, it is unlikely that the global dynamics of the transportation network would repeatedly undergo drastic changes between closely-taken snapshots from hour to hour. Therefore, we conclude that α provides a better and more informative picture of the network's evolution over time.

Also, note the approximately 10% drops in α at 8:00 and between 16:00-18:00 on weekdays (Supplementary Figure 6*D*) is consistent with circadian rhythm of urban human mobility, as these times are associated with morning and evening peak commuting periods, when high rates of congestion and large numbers of commuters predictably increase the conflict between PT system and road conditions.

We also tested the sensitivity of the two reliability indices (ρ_c and α), to the choice of parameter δ (time window length). The proposed α shows the same temporal trend in reliability of the network with different choices of δ for constructing the network structure and its corresponding flow demand matrix F (Supplementary Figure 6*C*&*D*). Overall, the results of our simulations suggest that the choices of δ from 1 to 3 hours do not have a significant impact on the temporal evolution of α and its consistency in a day-to-day comparison, or in other words, the daily trend is robust against alteration of δ within a wide range of reasonable values. Also, the relationship between α in weekday and weekend mode is robust against different choices of δ. Note that ρ_c is noticeably more sensitive to δ, and both its temporal trends during a day and the relation between its values in weekday and weekend modes change with different choices of δ.

There are also other facts about the actual Melbourne's on-road PT network which validate the results achieved by α and suggest that it works well. The relatively low reliability of the network during early morning and late evening is mainly due to fewer PT services running on the network which decreases the number of links and weakens the connectivity (Supplementary Figure 7). The reliability α is relatively stable over a day, despite multiple periods of intense traffic. This is partially explained by the fact that the average degree $\langle k \rangle$ is higher during the rush hours (Supplementary Figure 7*B*) indicating more available services or higher frequency of the existing services during those hours. Generally, larger number of links implies better connectivity which generally should increase the reliability α. If there were no additional PT services during rush hours (manifested by peaks in $\langle k \rangle$), we would see a larger drop in reliability α during rush hours. Thus, planning of the system contributes to stability of the network reliability α during the day by increasing the PT services around rush hours.

Supplementary Note 6: Evaluating the Bottlenecks of Melbourne's PT Network

Our proposed bottleneck identification approach leads to bottleneck links that are consistent with the formation of the hotspots in Melbourne urban area. Generally, there is a high passenger travel demand to and from important activity centers in urban areas, while due to traffic congestion and crowding, link-level quality of service can decrease to a great degree in the surroundings of such areas. Accounting for passenger flow demand, link dynamics, and structure of the network, the calculated link criticality scores were observed to be higher in proximity of known activity centers in Melbourne which is in agreement with the above facts (see Supplementary Figure 9*A*). Network bottlenecks were concentrated mainly around the Central Business District (CBD) and other commercial hubs in suburban areas. Bottlenecks associated with major university campuses outside CBD were among the top bottlenecks only on weekdays, which is interesting because universities are obvious hotspot points while they are only active during weekdays; compare left and right panels of Supplementary Figure 9*B*.

In order to assess the effectiveness of our identified bottlenecks in improving the network's reliability, we applied three well-established bottleneck identification methods on Melbourne's on-road PT network. We then compared the response of the network reliability to improving each of the four different types of bottlenecks. The bottlenecks identified based on our proposed approach are referred to as Criticality Score-based (CS) bottlenecks. The other three bottleneck identification approaches used in comparisons are explained below:

- Bottleneck identification based on Edge Betweenness centrality (EB bottlenecks): Edge betweenness centrality (9) of a link is defined as the number of shortest paths between all pairs of nodes in a network that traverse the given link. Betweenness centrality indicates the influence of the link on flow circulation when the optimal path for flow between a pair of nodes is assumed to be the shortest path on the network. Here, we used the normalized edge betweenness centrality calculated based hop count between the node pairs. The centrality of a link according to this measure, is the number of shortest paths between different O-D node pairs that the link is a part of; centralities are normalized by the total number of O-D pairs on the network. Separately for weekdays and weekends, links with the largest mean edge betweenness centrality scores (averaged over the two months of September and October 2017) were identified as EB bottlenecks of the network.

- Bottleneck identification based on Weighted Edge Betweenness centrality (WEB bottlenecks): Here, we also extend the standard edge betweenness centrality measure, to incorporate the flow-demand over the network when choosing the bottlenecks. In order to do so, the importance of the shortest path between each O-D node pair is weighted by the volume of the flow demand between that pair. The resulting scores of the links are then normalized by the total flow-demand over the network. The centrality of a link according to this measure is proportional to the volume of flow (i.e., number of passenger trips in our case study of a PT network) passing through the link as a part of the shortest path between their associated O-D pair of nodes. Thus, the links on the shortest path connecting a pair of nodes with higher flow demand volume become relatively more central. Separately for weekdays and weekends, links with the largest mean weighted edge betweenness centrality scores (averaged over the two months of September and October 2017) were identified as WEB bottlenecks of the network.

- Bottleneck identification based on Percolation Criticality (PC bottlenecks): Classical percolation-based reliability analysis views the links bridging between the clusters of higher quality links, as network bottlenecks (10, 11). In this approach, the key to bottleneck identification is in the study of the network at percolation criticality $\rho = \rho_c$, when the GC fragments into smaller sized components formed of links with quality higher than ρ_c. From the set of links removed at criticality (all of which have a quality equal to ρ_c), only a subset is actually responsible for the fragmentation of the GC (11). Let us refer to the set of all links removed at ρ_c as the candidate bottleneck set. The actual bottleneck links can be identified through an exhaustive search among all possible combinations of different number of bottlenecks in the candidate set, to discover the minimal subset that actually glue the GC together. However, this brute-force approach is impractical for a large candidate set. For Melbourne's on-road PT network, the size of the candidate set was over 100 during most of the times on weekdays. In order to identify the real bottlenecks, we counted the occurrences of each link in all candidate sets (associated with networks of different times) and the most frequently observed links were identified as the network bottlenecks.

Supplementary Note 7: Appendix to Generality of the Proposed Framework

Random Geometric Graph (RGG) structure (investigated in the *Main Text*) was the perfect choice for testing the generality of our framework as it shares common properties with many spatial infrastructure networks. Links on these networks either belong to relatively small well-connected local communities or they bridge between these communities. This allowed us to characterize our proposed bottlenecks which can shift toward either of the two above roles, depending on the flow demand distribution over the network. Here, we extend the analysis of different flow-demand scenarios to square grid and random network (ER) structures (Supplementary Figure 12). We analyzed the square grid and ER structures with three different flow-demand scenarios, namely, uniform, long-range, and short-range flow-demand. Unaffected Demand (UD) showed logical sensitivity to non-uniformity of flow-demand distributions on network.

Grid structure showed similar results to that of RGG when demand distribution changes between long-range, short-range, and uniform scenarios. These two structures, are both locally well-connected but do not have long-range links, thus, availability of more alternative paths between closely situated nodes makes them more reliable for serving short-range flows. In a grid

network structure, long-range (short-range) flows need to traverse more (less) links to reach destination thus they are more (less) likely to be affected by low-quality of links when link-qualities are distributed randomly over the network. This was reflected by UD with a faster (slower) decrease as a function of increasing ρ when flows tend to be long-range (short-range), resulting in a lower (higher) demand-serving reliability α compared to the uniform flow demand scenario; note the area under the curves marked by up (down) -pointing triangles in Supplementary Figure 12C, .

In ER networks, however, connectivity between nodes is independent from their Euclidean distance, thus, connectivity properties are similar between all pairs of nodes on the network. This, expectedly, resulted in an almost identical average UD_ρ over the number of realizations, for all three demand scenarios. Thus, the network is equally reliable when serving any of the three demand distributions.

Last but not least, for uniformly distributed flow demand over the network, the theoretical relationship $UD_\rho \approx (|GC_\rho|/n)^2$ (where n is the network size) between evolution of the Giant Component (GC) and UD as functions of the threshold ρ, is confirmed on ER and grid networks in addition to RGG which was studied in the *Main Text*; compare the blue curve and the dashed black curve in Supplementary Figure 12$C\&F$.

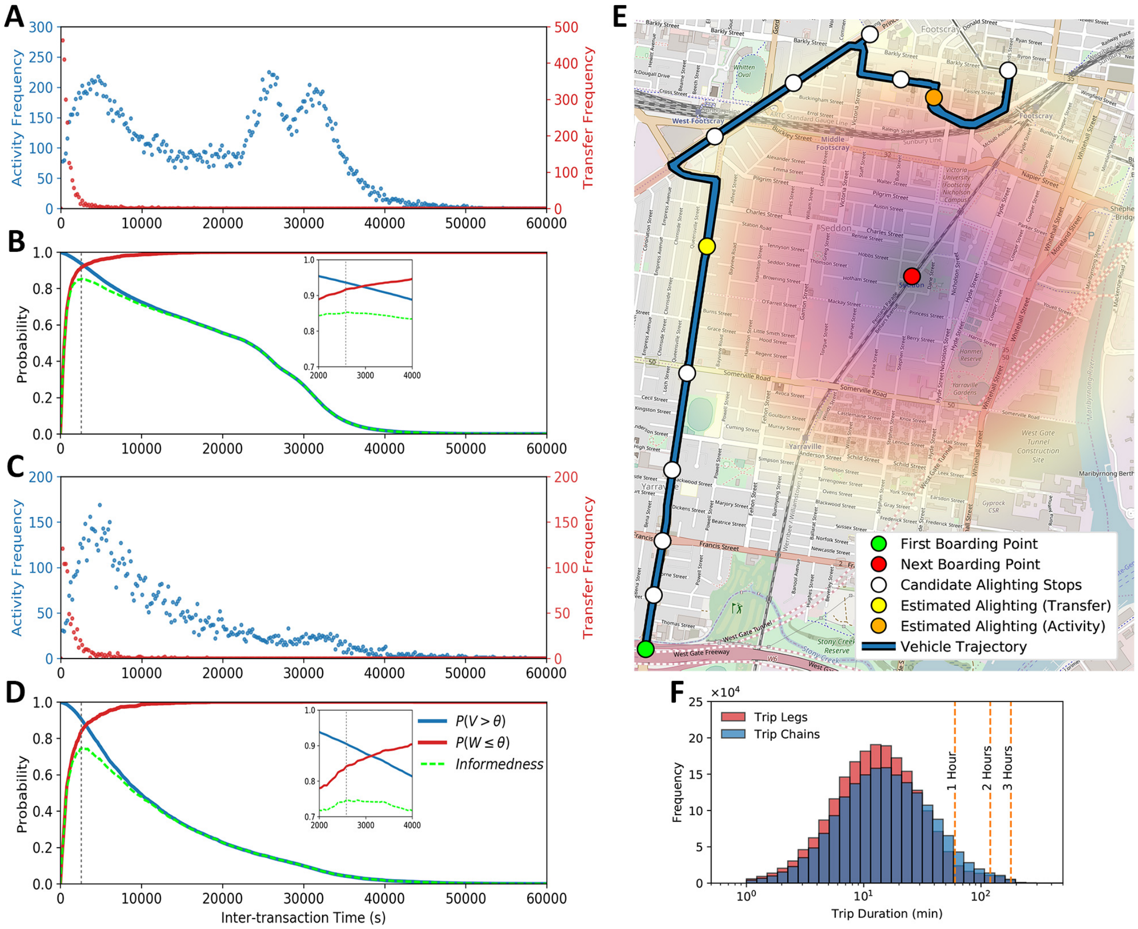

Supplementary Figure 1. Smart-card data processing. (*A*&*C*) Distribution of activity duration (blue) and transfer duration (red) within first five working days (*A*) and first five weekend days (*B*), in September 2017. (*B*&*D*) The cumulative distribution function (CDF) of transfer duration (red curve) and complementary cumulative distribution function (CCDF) of activity duration (blue curve) on workdays (*B*) and weekends (*D*). *Informedness* (green dashed curve) of the CDF and CCDF manifests the accuracy of discerning activities from transfers for different allowable transfer times. (*E*) An example of estimating a missing alighting transaction, where a boarding transaction (green) on a bus misses a valid scan-off pair. However, a scan-on transaction (red) is recorded for the same smart-card identifier later at a train station, with a location and timestamp that allows a non-empty set of plausible alighting stops (white). Among the candidate bus visits, the visited stop with the smallest Euclidean distance to the next boarding point, and the visited stop leading to the earliest arrival of the passenger to the next boarding location, are colored orange and yellow, respectively. Radial color gradient is depicted to aid comparing the distances from the final boarding stop to different candidate stops. Street map layer © OpenStreetMap contributors (12). (*F*) Histogram of single trip-leg (red) and O-D trip (blue) duration during September 2017.

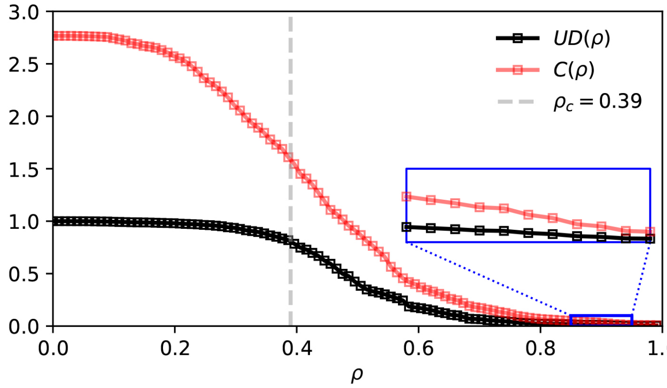

Supplementary Figure 2. Monitoring both the unaffected demand and capacity of the network under percolation. The plot depicts the percolation process on one snapshot (at 8:00 AM on 1 September 2017) of the Melbourne's public transportation (PT) network monitored by $UD(\rho)$ (same as in Fig. 2d of the *Main Text*); the percolation critical point is at $\rho_c = 0.39$. The red curve shows the capacity $C(\rho)$ of the subnetwork G_ρ at different thresholds ρ during the percolation process. For both $UD(\rho)$ and $C(\rho)$, units are relative to the total flow-demand on the network, that is UD at threshold $\rho = 0$ ($UD(0)$) when no link is removed. The inset shows that the capacity remains above the demand at the end of the percolation process.

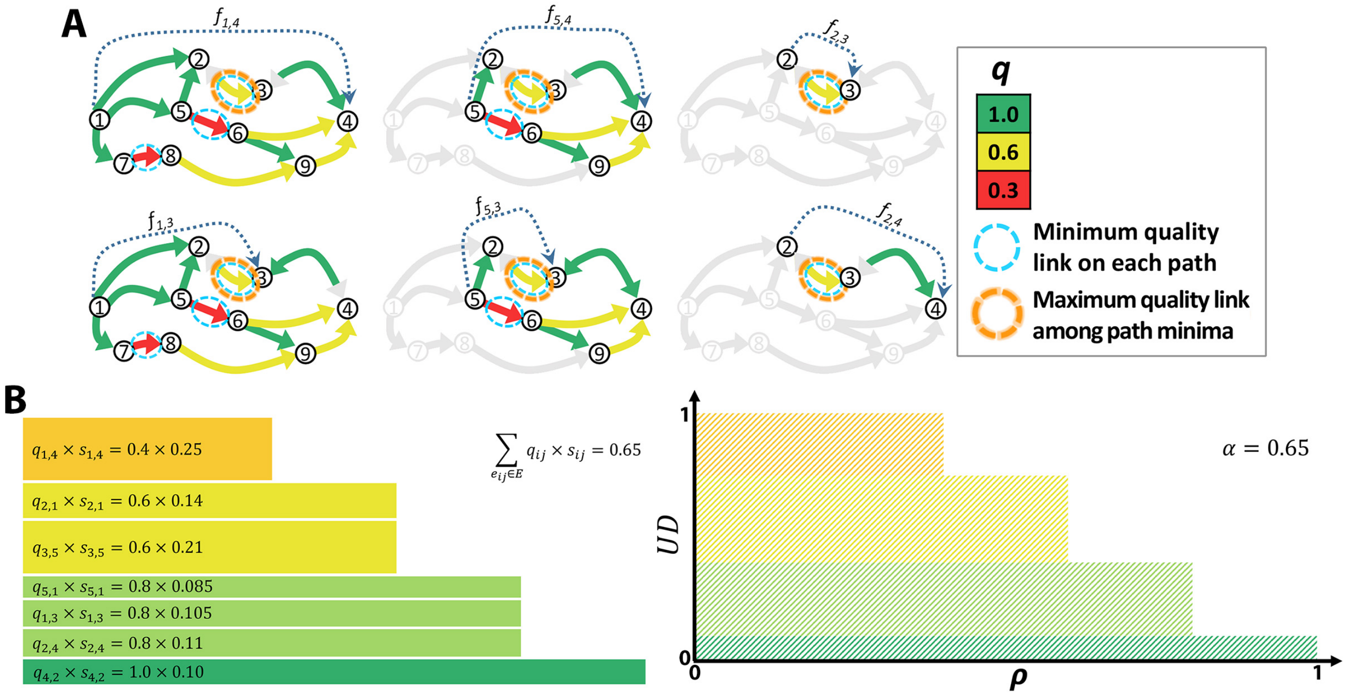

Supplementary Figure 3. Visualization of link criticality score calculation and its relationship with link quality and network reliability. (*A*) In the example provided in Fig. 3 (of the *Main Text*), the "limiting link" associated with the node pair $(1, 4)$ is found to be $e_{2,3}$; i.e. $e^*_{1,4} = e_{2,3}$. However, to calculate the criticality score of a link we need to know all pairs of origin-destination nodes between which the link acts as the limiting link. In the toy network of Fig. 3a (*Main Text*), $e_{2,3}$ is the limiting link for six different (o, d) node pairs, i.e., $e^*_{1,4} = e^*_{5,4} = e^*_{2,3} = e^*_{1,3} = e^*_{5,3} = e^*_{2,4} = e_{2,3}$. In *A*, each of those (o, d) pairs is indicated with a dashed arrow from node o to d on a separate copy of the network, where the links which are not part of the paths connecting o to d are colored light gray. By definition of link criticality score, $s_{2,3}$ is the sum of flow demand between these node pairs, divided by the total flow demand on the network. (*B*) Illustration of relationship between the reliability α, link qualities q_{ij}, and link criticality scores s_{ij} for the network in Fig. 1a of the *Main Text*. On the left, link quality multiplied by link criticality score is visualized for network links with non-zero criticality scores. Links are sorted in ascending order of their quality from top to bottom. On the right, the area under curve of Unaffected Demand (UD) as a function of the threshold ρ (which is seen in Fig. 1b of the *Main Text*) is partitioned into multiple rectangles (each corresponding to a network link) with different colors to show the relationship between the proposed reliability α, link qualities q_{ij}, and link criticality scores s_{ij}.

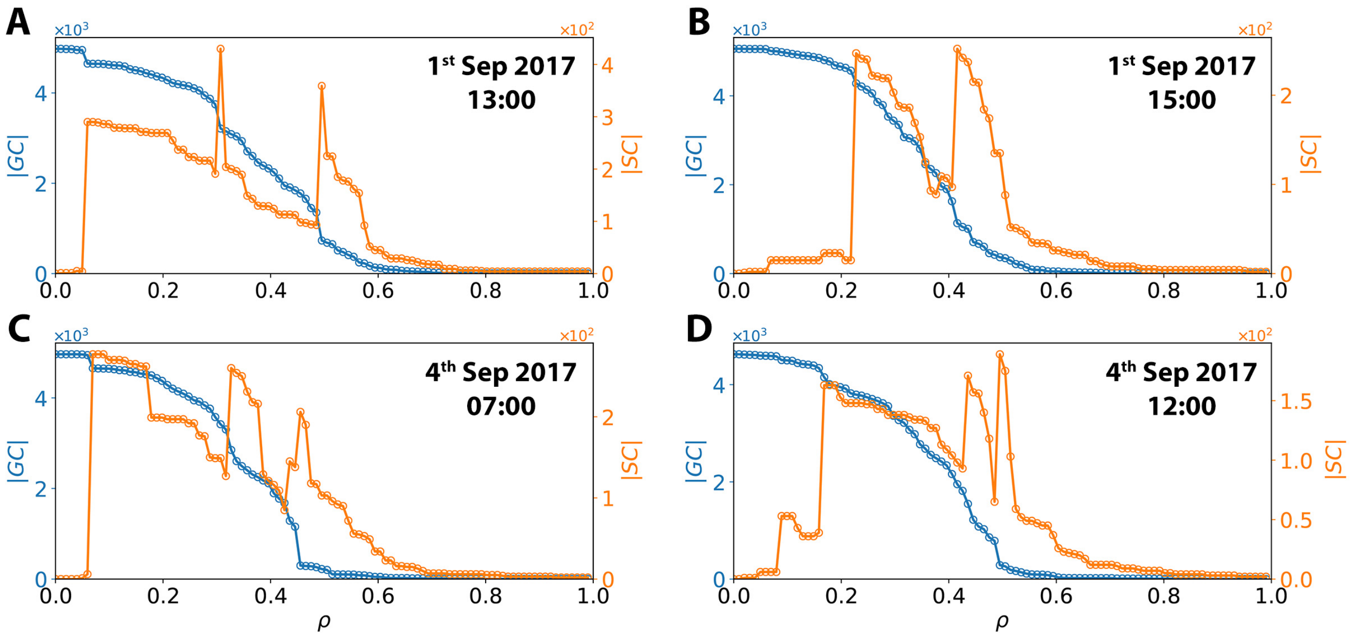

Supplementary Figure 4. Absence of a single abrupt phase transition. Four examples are illustrated from Melbourne's Public Transportation (PT) network from the first two weekdays of September 2017, where it is difficult to find the critical threshold ρ_c. Evolution of $|GC|$ (blue) and $|SC|$ (orange) as functions of ρ are depicted for Melbourne's on-road PT network at (*A*) 13:00 on 1st, (*B*) 15:00 on 1st, (*C*) 7:00 on 4th, and (*D*) 12:00 on 4th of September 2017. In *A* and *C* the three peaks in $|SC|$ demonstrate the detachment of a component of substantial size from the GC at least at three different thresholds. However, none of the three fragmentations is highly distinctive from the rest, and the most severe reduction in $|GC|$ does not happen at the point of maximum $|SC|$. In *B*, the two peaks in $|SC|$ have only 5 nodes difference, yet the percolation criticality is marked by the second peak, which occurs at a threshold ρ approximately 0.2 higher than the first one. During the percolation shown in *D*, $|SC|$ always remains insignificant (< 200) relative to $|GC|$ (> 4500), which suggests that practically the percolation process only gradually erodes the GC and the fragmentation is blurred out over a range of ρ values. These examples demonstrate that at times there is no clear fragmentation of the GC at a single threshold on the Melbourne PT network, due to its finite size and non-random character.

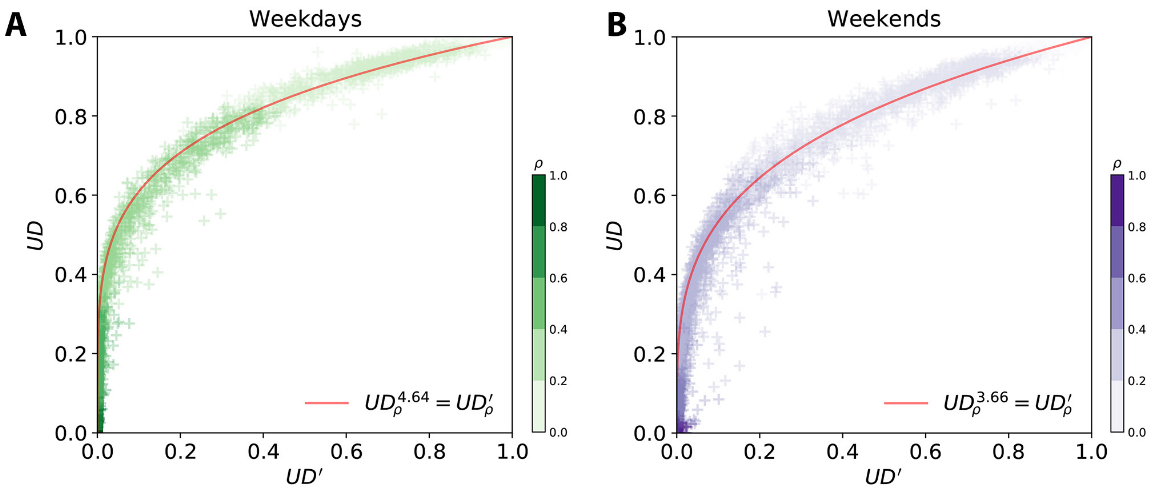

Supplementary Figure 5. Capturing the heterogeneity of flow demand via Unaffected Demand (UD). UD on Melbourne's PT network during the percolation process, in the presence of actual travel flow demand versus a synthetic uniform travel flow demand, during (*A*) weekdays and (*B*) weekends. Data points show the results for networks of the first two weeks in September 2017, i.e. 148 networks for weekends and 370 networks for weekdays. For network of each particular time, 26 data points are scattered for threshold ρ values (depicted by color intensity) between 0 and 1 with steps of 0.04. With increasing ρ, UD' (the proportion of connected node pairs) decreases faster than UD (the proportion of unbroken trips). Furthermore, the decrease in UD' is faster in weekdays compared to that of weekends.

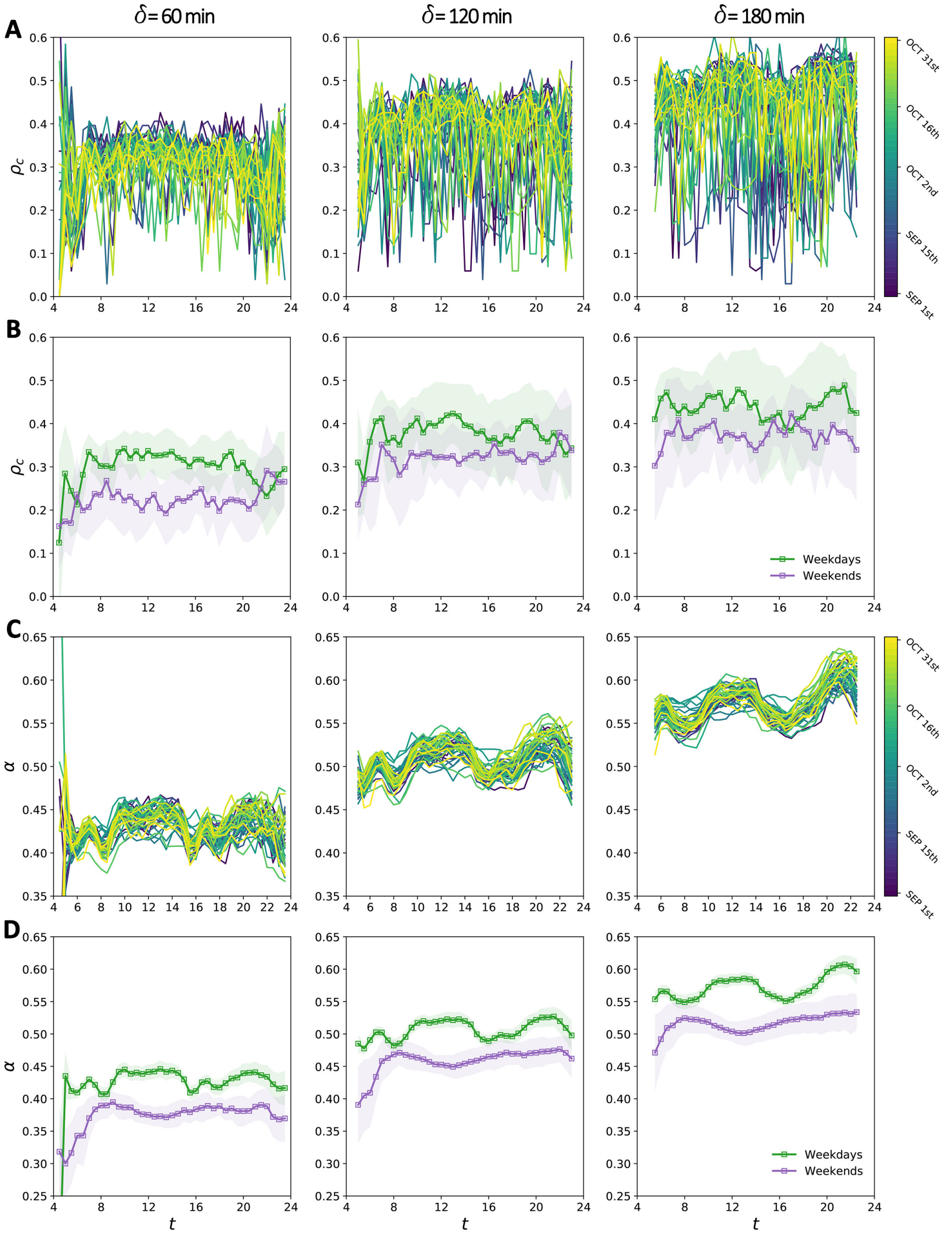

Supplementary Figure 6. Temporal reliability of Melbourne's on-road Public Transportation (PT) network. Temporal evolution of reliability indices ρ_C and α during the day is depicted for Melbourne's PT network with δ set as 60 min (left column), 120 min (middle column), and 180 min (right column) while time window is moved in 30 min steps over the day. Temporal evolution of (*A*) ρ_c and (*C*) α, during the day for all weekdays (each day has a unique color) in September and October 2017. Mean (*B*) ρ_c and (*D*) α, as a function of time of the day, averaged separately over weekdays (green) and weekends (weekends). In *B* and *D*, thickness of the shaded area around the curve is equal to two standard deviations at each particular time of the day.

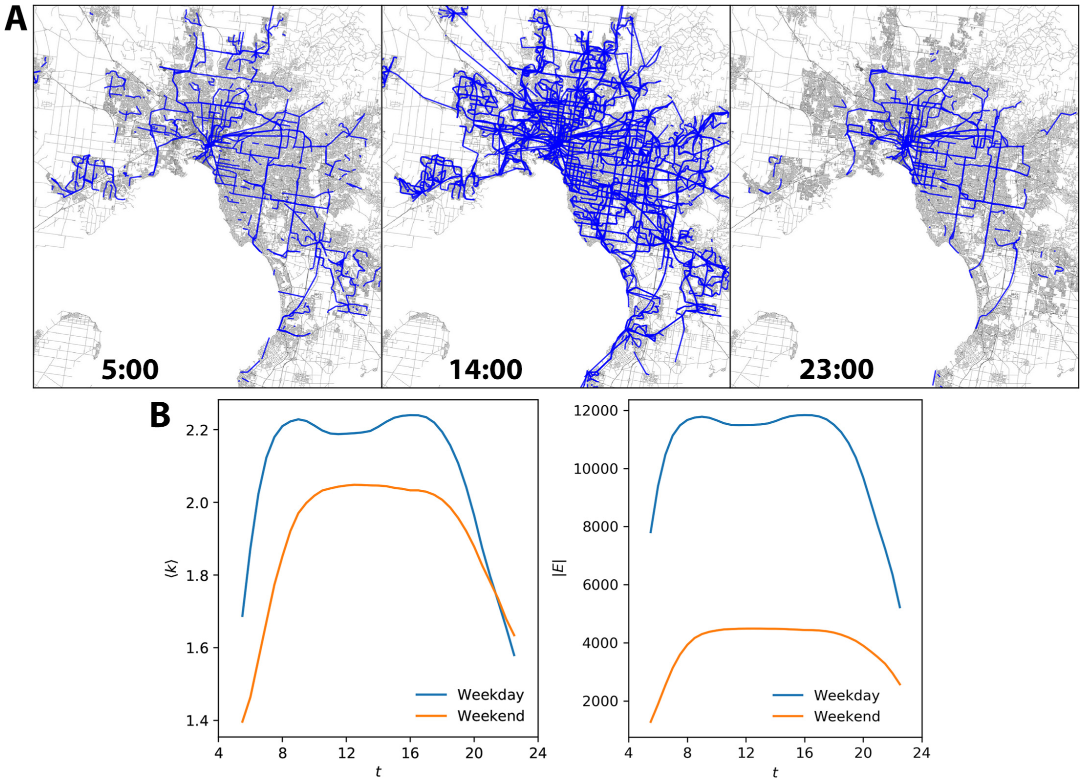

Supplementary Figure 7. Temporal structure of Melbourne's on-road Public Transportation (PT) network. (*A*) The maps show the structure of the network at three points in time over a normal weekday. Each link on the network is depicted with a straight blue line connecting its source and target nodes (corresponding to stops). Street map layers © OpenStreetMap contributors (12). (*B*) Temporal average degree $\langle k \rangle$ of the network and the number of its links $|E|$ versus time t of the day, separately for weekdays (blue) and weekends (orange).

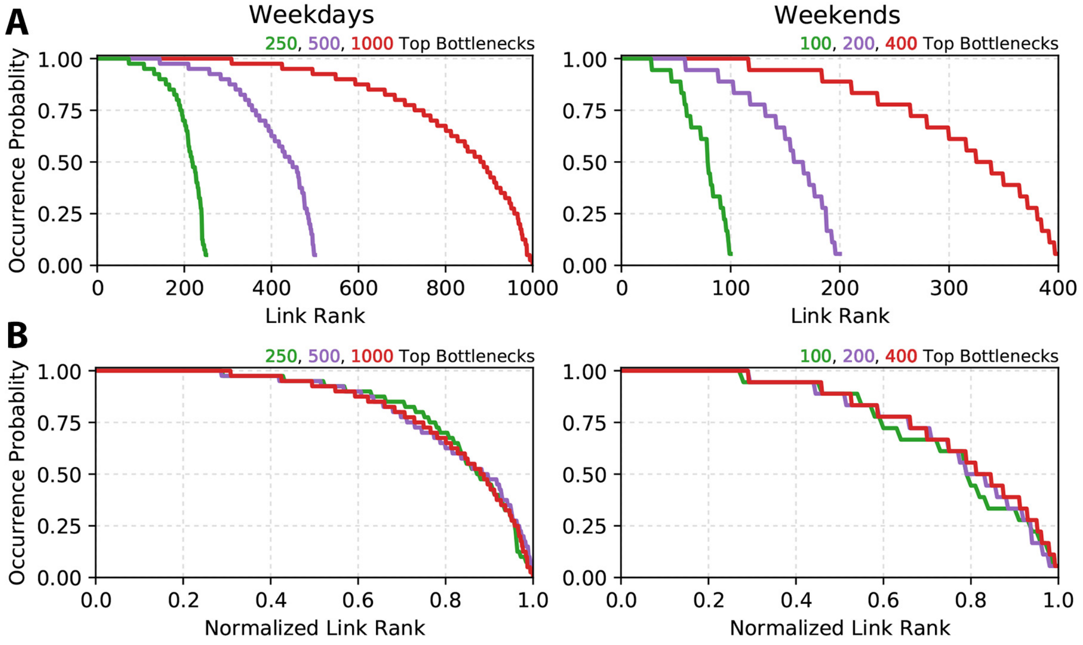

Supplementary Figure 8. Robustness of the identified bottlenecks in Melbourne's PT network. Here, we investigate the extent to which the identified network bottlenecks, persist as the most critical links on the networks of different days. So, we calculated the average criticality score of each link over different times of each particular day to identify the most critical link on the day. Then, we counted the number of the occurrences for each identified bottleneck among the top most critical links of each day. (*A*) Fraction of days on which top bottlenecks appear in the set of most critical daily links versus the link rank while links are sorted in descending order of their robustness over the different. (*B*) Same as *A*, but the link ranks are normalized between 0 and 1 (most and least persisting links are associated with 0 and 1 respectively) to show that the results are not sensitive to the number of selected bottlenecks. In separated experiments, the number of top bottlenecks of the network and most critical links on each day are both limited to 250 (100), 500 (200), and 1,000 (400) for networks of weekdays (weekends). Regardless of the number to which we limit number of to daily critical links and network bottlenecks, almost 80% of the top bottlenecks, appear as the most critical link on approximately 75% of the days, supporting the robustness of the identified network bottlenecks.

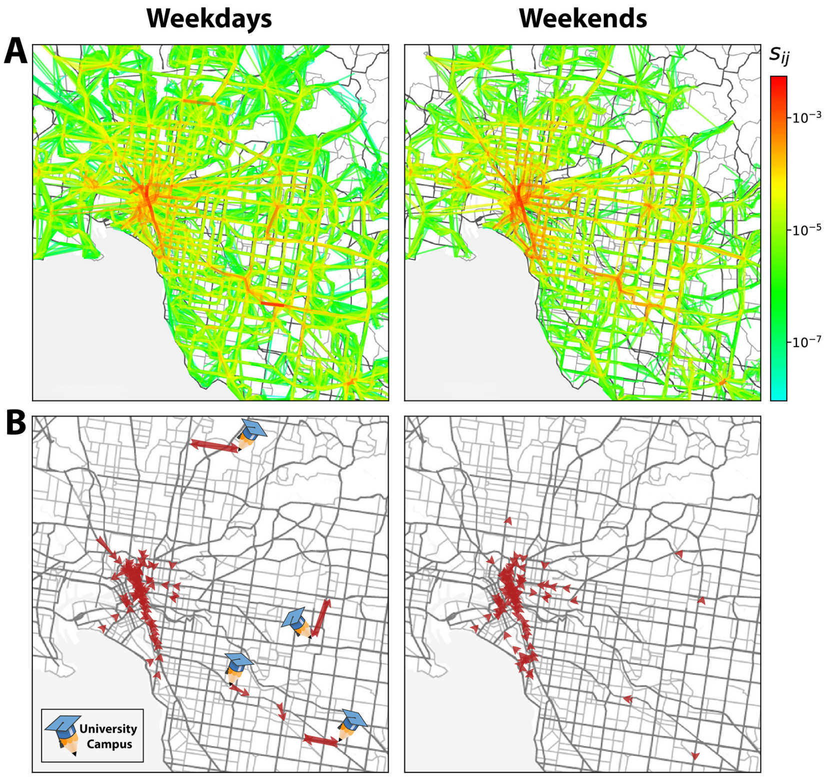

Supplementary Figure 9. Spatial distribution of critical links. (*A*) Spatial distribution of the link criticality scores over the Melbourne's on-road PT network, separately for weekdays (left panel) and weekends (right panel). (*B*) Top 100 bottlenecks identified according to link criticality scores, separately for weekdays (left) and weekends (right). The major visible distinction between the top 100 bottlenecks of weekdays and weekends is emergence of the links associated with major university campuses (pinned on the map) outside Melbourne CBD area on weekdays. This is an evidence on alignment of the identified bottlenecks with urban activity centers, as large university campuses are obvious hotspot points during the weekdays which cease to act as hotspots during the weekends. Street map layers © OpenStreetMap contributors (12).

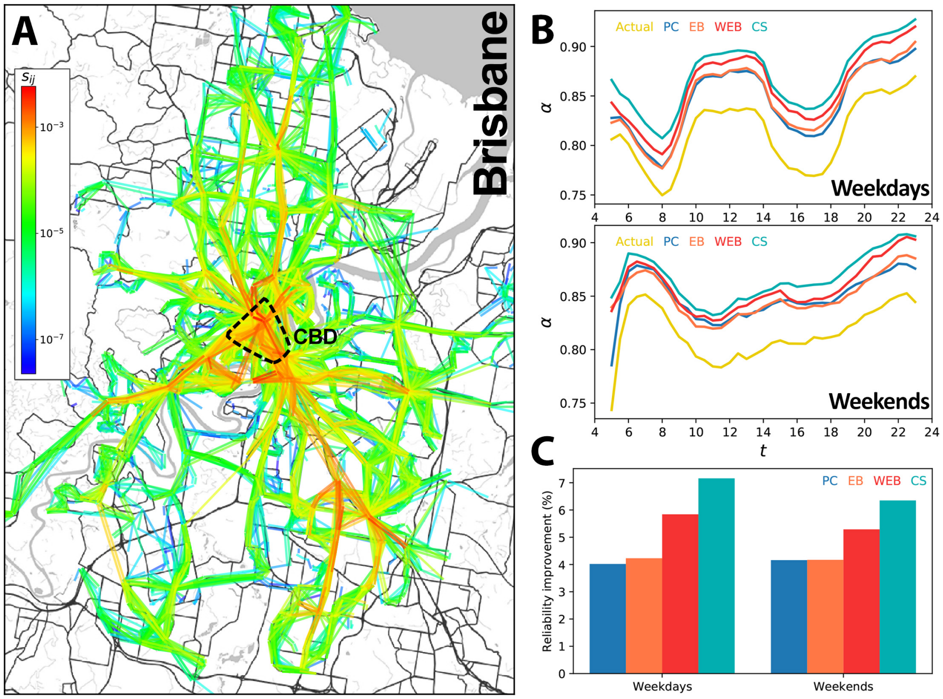

Supplementary Figure 10. Improving Brisbane's on-road public transportation network. (*A*) Spatial distribution of the link criticality scores over the Brisbane's on-road PT network of weekdays. Street map layer © OpenStreetMap contributors (12). (*B*) Reliability α over the day calculated for the actual (yellow) network, and its synthetically improved versions obtained by ameliorating CS bottlenecks (cyan), EB bottlenecks (orange), WEB bottlenecks (red), and PC bottlenecks (blue); each curve shows the average over the days of March 2013. (*C*) Overall improvement in network reliability α achieved by amelioration of different types of bottlenecks; the results correspond to the average of α over all snapshots of the network, separately, during weekdays and weekends.

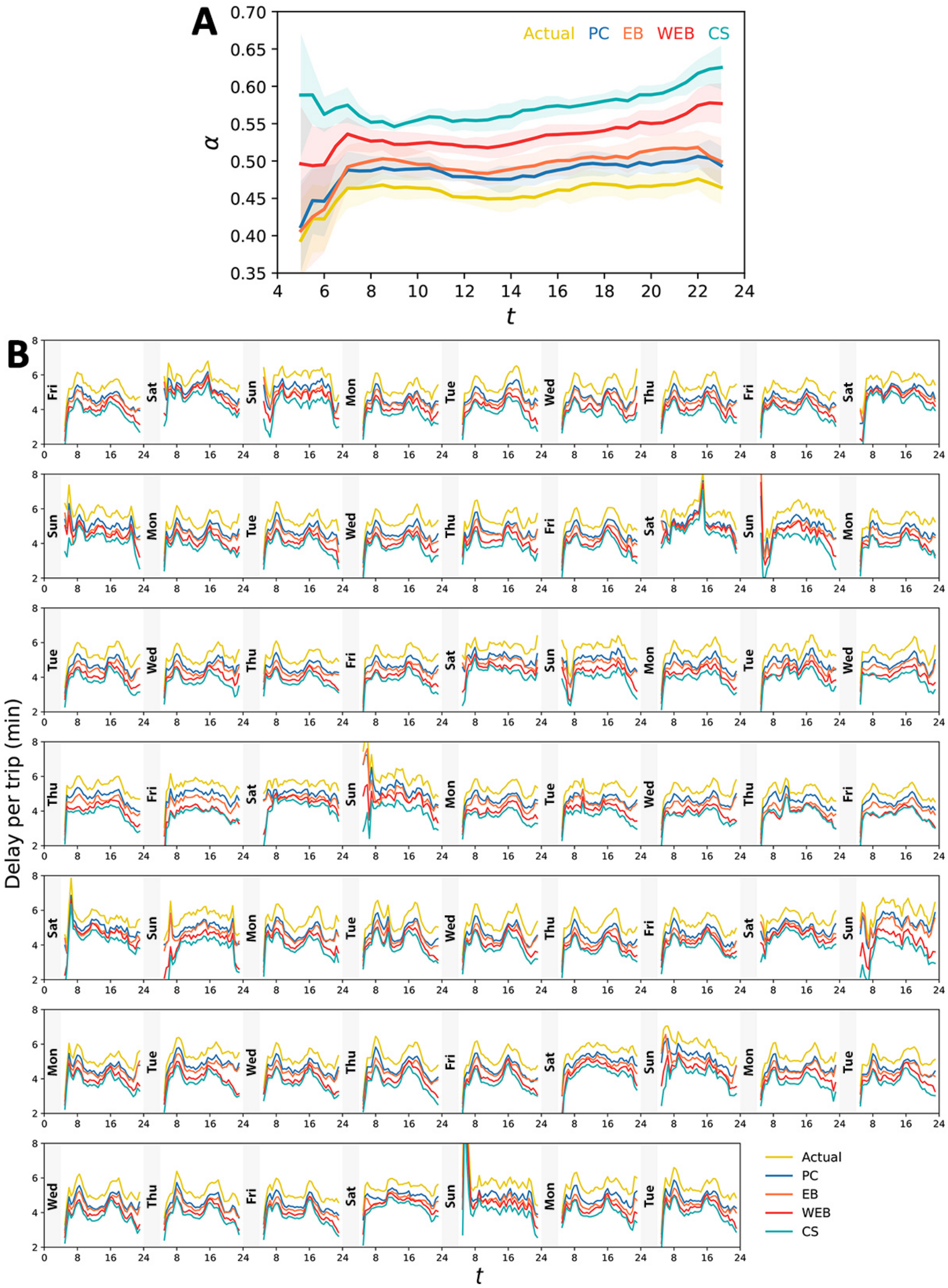

Supplementary Figure 11. Improving Melbourne's Public Transportation (PT) network by ameliorating its bottlenecks. (*A*) Daily evolution of α calculated for the actual (yellow) and improved networks obtained by ameliorating CS bottlenecks (cyan), EB bottlenecks (orange), WEB bottlenecks (red), and PC bottlenecks (blue). Results show the average (solid line) and standard deviation (shaded area) over the weekend days during September and October 2017. (*B*) Effect of improving bottlenecks on the delay caused by road conditions. Additional delay per passenger trip imposed by the road conditions on the PT system is shown for the actual network and improved networks. Improved networks are simulated by synthetically increasing the quality of bottlenecks identified using three well-established approaches in addition to our proposed approach.

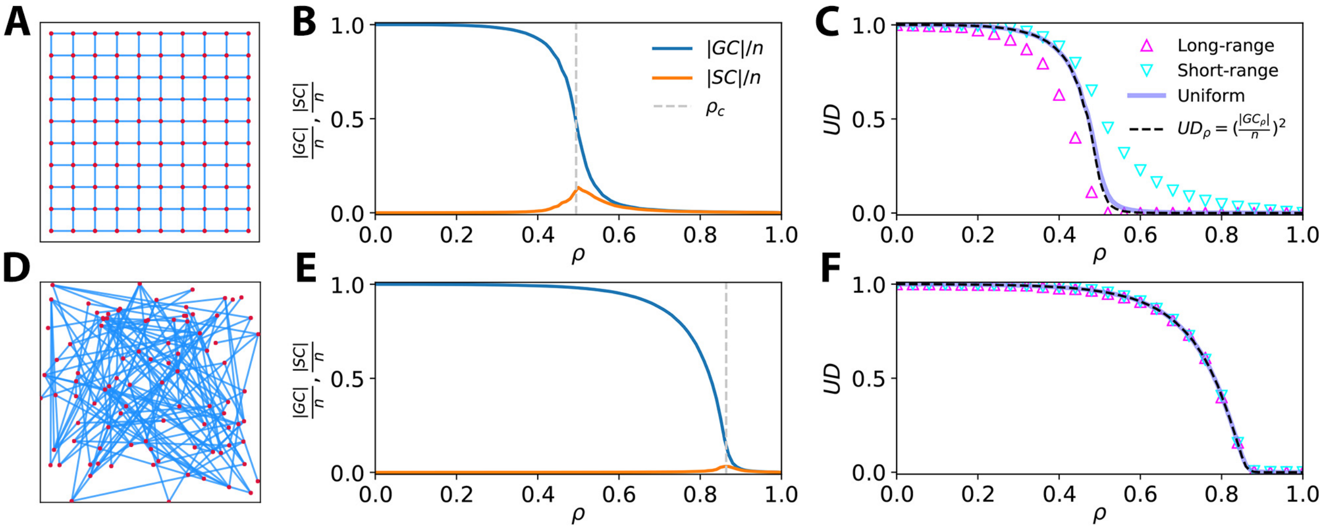

Supplementary Figure 12. Capturing properties of the demand-serving networks with grid and random graph structure. (*A*) A sample square grid graph. (*B*) Normalized $|GC|$ and $|SC|$ during the percolation process averaged over 100 realizations of random link qualities on a square grid of size 2,500 nodes. (*C*) Unaffected Demand (UD) versus ρ for different flow demand scenarios on the grid structure, averaged over 100 realizations. (*D*) A sample random graph generated using Erdős-Rényi (ER) model (13) where nodes are attributed with a random spatial position. (*E*) Normalized $|GC|$ and $|SC|$ during the percolation process averaged over 100 realizations of random link qualities on ER networks of size 2500 and average degree of $\langle k \rangle \approx 8$. (*F*) UD versus ρ for different flow demand scenarios on ER network structures, each averaged over 100 realizations.

Supplementary References

1. School policy and advisory guide: School hours. (https://www.education.vic.gov.au/school/principals/spag/management/Pages/hours.aspx) (2019) [Accessed 21/07/2020].
2. Fair work ombudsman: Full-time employees. (https://www.fairwork.gov.au/employee-entitlements/types-of-employees/casual-part-time-and-full-time/full-time-employees) (2019) [Accessed 21/07/2020].
3. WJ Youden, Index for rating diagnostic tests. *Cancer* **3**, 32–35 (1950).
4. A Alsger, B Assemi, M Mesbah, L Ferreira, Validating and improving public transport origin–destination estimation algorithm using smart card fare data. *Transp. Res. Part C: Emerg. Technol.* **68**, 490–506 (2016).
5. M Munizaga, F Devillaine, C Navarrete, D Silva, Validating travel behavior estimated from smartcard data. *Transp. Res. Part C: Emerg. Technol.* **44**, 70–79 (2014).
6. LK Fleischer, Approximating fractional multicommodity flow independent of the number of commodities. *SIAM J. on Discret. Math.* **13**, 505–520 (2000).
7. M Pollack, Letter to the editor—the maximum capacity through a network. *Oper. Res.* **8**, 733–736 (1960).
8. EW Dijkstra, A note on two problems in connexion with graphs. *Numer. Math.* **1**, 269–271 (1959).
9. M Girvan, ME Newman, Community structure in social and biological networks. *Proc. Natl. Acad. Sci.* **99**, 7821–7826 (2002).
10. YN Kenett, et al., Flexibility of thought in high creative individuals represented by percolation analysis. *Proc. Natl. Acad. Sci.* **115**, 867–872 (2018).
11. D Li, et al., Percolation transition in dynamical traffic network with evolving critical bottlenecks. *Proc. Natl. Acad. Sci.* **112**, 669–672 (2015).
12. Openstreetmap copyright and license. (https://www.openstreetmap.org/copyright) (2019) [Accessed 21/07/2020].
13. B Bollobás, B Béla, *Random graphs.* (Cambridge university press) No. 73, (2001).
