# Supplementary material for: Percolation of heterogeneous flows uncovers the bottlenecks of infrastructure networks
Source: Nat Commun. 2021 Feb 23;12:1254. doi: 10.1038/s41467-021-21483-y (PMC7902621; doi:10.1038/s41467-021-21483-y)
Supplement: Supplementary file 2 — Peer Review File [file 41467_2021_21483_MOESM2_ESM.pdf]

Reviewer #1 (Remarks to the Author):

Report on the manuscript entitled "Percolation of heterogeneous flows uncovers the bottlenecks of infrastructure networks" by Homayoun Hamedmoghadam et al.

The authors propose a variant of a percolation-based transportation network analysis that accounts for public traffic flow heterogeneity. The authors argue that their approach is superior to conventional percolation-based approaches to traffic analysis. As an application of the percolation of heterogeneous flows the authors suggest the identification of transportation bottlenecks scheme.

The development and support of efficient, resilient and sustainable transportation infrastructure is on the top of the agenda in most developed and developing nations, making the subject of this study definitely worth the investigation, and well within the scope of Nature Communications.

The manuscript is well written, adequately explained and presented at the level accessible not only to specialists but also to general readership of the journal. While the presented material is certainly novel, I have certain concerns about the potential impact of the findings and the conclusions made in the manuscript.

1. On the conceptual level, the authors propose a demand-serving reliability index \alpha as the area under the unaffected demand (UD) curve. While the proposed index indeed characterizes traffic reliability, there are multiple possible ways to define it. One alternative way, for instance is to define \alpha as the area under the curve of the UD^{\tau}, where \tau>0 could be any constant exponent. In case of homogeneous flows and \tau = 1/2 the proposed measure would correspond to the proposed in earlier works size of the giant connected component.

I am wondering if it is possible to argue that the proposed definition of \alpha is the only one or the best one to characterize traffic reliability.

2. Bases on the quality attribute index the authors derive the link criticality score to highlight critical transportation links in the Melbourne’s bus and tram Public Transportation (PT) network. Both as a reader and a reviewer I am wondering if it is possible to assess the accuracy of link criticality score in a direct way?

Is there a ground-truth data for the most critical transportation links?
Can authors argue that the improvement of transportation links according to the link criticality score is more efficient than that according to alternative measures, say, betweenness centrality?

On the technical level:

3. The quality attribute q_ij depends on travel times along link ij, which are determined by congestion levels and should strongly depend on the network structure.
When one performs percolation, network links are removed and travel time along each surviving link should be updated. Since quality attribute values are functions of these
times, should not quality attributes be updated at each step of the percolation procedure?

I invite authors to clarify this aspect in the main text. In case, this question has already been answered in previous studies, I invite the authors to provide relevant references.

4. I invite authors to extend the analysis to cities other than Melbourne. In the current form, the manuscript is only analyzing traffic in one city and it is not clear how general the findings are. Tests on synthetic random geometric graphs only partially resolve the problem due to the uniform traffic patterns.

5. When modeling random geometric graphs, the authors set the connectivity parameter of r_0 to 1.5. How is this parameter selected?

In summary, the present manuscript contains interesting findings that might have significant impact on our understanding of transportation efficiency. Since this impact is not sufficiently well established, I cannot recommend the publication of the manuscript in Nature Communications in its present form. A careful revision of the manuscript according to my comments might make the manuscript publishable in the future. My recommendation stands as "revise and resubmit".

Reviewer #2 (Remarks to the Author):

This study represents a very good interdisciplinary research of transportation science and network science. The match between traffic demand and network percolation is of great concern for traffic management. However, considering the complexity of large transportation networks, the lack of effective theories and techniques is the "pain point". Based on real smart-card data, the authors applied a percolation-based method to analyze the public transportation reliability of a typical infrastructure network – the public transportation system in Melbourne. The authors introduced a demand-serving reliability (i.e., based on Unaffected Demand) which is the combination of flow demand and traffic percolation. Within this framework, the road affecting much the flow demand can be viewed as the "bottleneck". Furthermore, the authors uncovered how the identified bottlenecks can improve the whole traffic.

Percolation theory has recently been widely applied to analyze the properties of the traffic network. However, to the best of my knowledge, this work is original and interesting to apply in the real flow demand in traffic percolation. The authors' definition of demand-serving reliability is very insightful and practical for traffic management.
Based on their framework, they build up a tie between the microscopic link importance (bottlenecks) and the macroscopic system performance (reliability) in a simple but effective way as manifested by Eq. (5), which is one of their key results. Indeed, improvement of a few bottlenecks identified by their methods can significantly influence the global network performance, which is demonstrated by real data testing. It is an inteesting perspective to consider the relationship between flow distribution and its traffic percolation process. With implications in both model networks and real-world networks, the authors have demonstrated that the uniformly distributed flow network shows similar percolation properties to the classic percolation model. This finding can shed light on future traffic management strategies – like by adjusting the locations of city functional regions to generate more OD paths with short distance and large flow.

Considering its novel and noteworthy findings, as well as the significant contributions it makes to the related fields, I believe that this paper fits perfectly the publication standard required by Nature Communications.

Before publication, I would like to ask the authors to clarify some of the following points:

(1) The authors discuss well the percolation of public transportation. In this sense, I wonder if the multiple paths identified by the authors between the same pair can be compared in time scale. This

could of course influence the travel experience of each user.

(2) Following the above question, when a given path is destroyed, can the alternative paths between the same OD pair match the amount of total traffic demand? Will some paths left influence the cascading failure effect?

(3) In Fig. 2, authors claim that at percolation critical point, the unaffected demand is still 80%. Is this calculation consider the flow capacity of the giant component?

(4) The traffic reliability alpha is found larger in weekdays than in weekends as shown in FIG. 2e. The authors argue that this may be due to the impact “by lower-quality links during weekends compared to weekdays” . Can the authors give some examples of specific roads? Because when comparing the spatial distribution of critical links (as shown in FIG. S8A), it looks very similar between weekdays and weekends to readers.

(4) In line 70-71, the authors tell us the total flow demand of on-road PT in weekdays and weekends, where a big volume gap exists (470,000 - 210,000 = 260,000 > 210,000). Generally, if the flow demand increases so much, the network reliability culd have hard time to stay in a higher level. However, the results show that in weekends the network reliability is much lower than in weekdays. The effectiveness of the reliability measure may depend on how much the PT takes up in the whole on-road transportation system. Can the authors discuss and clarify this issue?

Reviewer #1 (Remarks to the Author):

Report on the manuscript entitled "Percolation of heterogeneous flows uncovers the bottlenecks of infrastructure networks" by Homayoun Hamedmoghadam et al.

The authors propose a variant of a percolation-based transportation network analysis that accounts for public traffic flow heterogeneity. The authors argue that their approach is superior to conventional percolation-based approaches to traffic analysis. As an application of the percolation of heterogeneous flows the authors suggest the identification of transportation bottlenecks scheme.

The development and support of efficient, resilient and sustainable transportation infrastructure is on the top of the agenda in most developed and developing nations, making the subject of this study definitely worth the investigation, and well within the scope of Nature Communications.

The manuscript is well written, adequately explained and presented at the level accessible not only to specialists but also to general readership of the journal. While the presented material is certainly novel, I have certain concerns about the potential impact of the findings and the conclusions made in the manuscript.

Response: We thank the reviewer for these nice comments and useful suggestions which have led to overall improvement of the manuscript, and especially helped establishing the impact of the study.

----

1. On the conceptual level, the authors propose a demand-serving reliability index \alpha as the area under the unaffected demand (UD) curve. While the proposed index indeed characterizes traffic reliability, there are multiple possible ways to define it. One alternative way, for instance is to define \alpha as the area under the curve of the UD^{\tau}, where \tau>0 could be any constant exponent. In case of homogeneous flows and \tau = 1/2 the proposed measure would correspond to the proposed in earlier works size of the giant connected component.

I am wondering if it is possible to argue that the proposed definition of \alpha is the only one or the best one to characterize traffic reliability.

Response: The reviewer is correct that defining α as the area under the curve of UD^{τ} with $\tau = 1$, is not the only possible approach. Different choices for $\tau > 0$ could be used for specific purposes. (The notation for the parameter τ is proposed by the reviewer in their comment, and we continue using it in this response, although it is used to denote travel time in the manuscript.) As noted and mentioned by the reviewer, in the manuscript we showed that using $\tau = 1/2$ the existing percolation paradigm becomes a special case of UD^{τ} for the restricted scenario where flow-demand is homogeneous. The flexibility of UD comes from the idea of keeping track of "connected node pairs", but the conventional percolation keeps track of "connected nodes," which is why τ turns out to be equal to 1/2 ($|nodes| \propto |node\ pairs|^{1/2}$) when relating the two approaches. However, to define α from UD^{τ}, we believe that our original choice of $\tau = 1$ in the manuscript is the natural choice as it preserves the association between α and "node pairs". This is probably the best choice leading to logical and simple relationships between the different variables in our theoretical framework (e.g., what we found by Eq. (5)), whereas any other scaling makes the relationships very complex and hard to utilize.

Below we provide more technical details on the above points, although the reviewer could skip this next section without loss of continuity:

i) Reliability α captures how the movement of each unit of flow demand is limited by a certain level of congestion on the network, and if $\tau = 1$ (as we set it in the paper), then each unit of flow demand will have the same weight in determining α. However, if $0 < \tau < 1$ (or $\tau > 1$) then a unit of demand affected by higher levels of congestion will have relatively more (or less) weight in the aggregation (i.e., reliability α) compared to those affected by lower levels of congestion. This is because $UD^{\tau}(\rho)$ is a monotonically decreasing function of ρ (representing congestion level), and by taking $0 < \tau < 1$ (or $\tau > 1$), the decrease in $UD^{\tau}(\rho)$ becomes understated (or exaggerated). We believe that $\tau = 1$ is a logical choice so that α represents the average congestion level limiting (or affecting) the movement of a unit of flow demand over the network.

ii) Different levels of congestion represented by link quality q (corresponding to ρ in the percolation process) have a linear relationship with link travel time (or velocity). Reliability α defined based on UD^{τ} preserves this linear relationship if τ is set to unity. And this allows for the mathematical conditions that lead to the simple relationship between link criticality scores, their quality, and network's reliability, i.e., $\alpha = \sum_{e_{ij} \in E} q_{ij}.s_{ij}$ (Eq. (5) in the *Main Text*). If $\tau \neq 1$, then the simple formula of Eq. (5) does not hold anymore. The relationship represented by Eq. (5) is fundamental to our framework, as it states: (loosely speaking) increasing the quality of a link with higher criticality score (s), leads to higher improvement on reliability of the network (α).

Action: We have stated in the revised text that other scalings of UD can also be used in the definition of reliability α (see "Methods"). We have also highlighted that our choice of $\tau = 1$ makes the theoretical analysis far simpler than any other option.

---

2. Bases on the quality attribute index the authors derive the link criticality score to highlight critical transportation links in the Melbourne's bus and tram Public Transportation (PT) network.

2.1 Both as a reader and a reviewer I am wondering if it is possible to assess the accuracy of link criticality score in a direct way?

Response: We thank the reviewer for this excellent question, which motivated us to design a direct analysis of our bottleneck identification accuracy. Before we present the new analysis (in the "Action" section below) added to the revised manuscript, we would like to point out an existing analysis in our original paper which was intended to demonstrate the accuracy of link criticality scores, in the section "Generality of the proposed framework." Through a controlled experiment, we investigated whether or not the calculated link criticality scores are consistent with the apparent "ground truth." For this purpose, we used RGG networks, where, loosely speaking, links can be categorized into those which lie inside well-connected local communities and those which bridge between the communities (light- vs. dark-colored links in Fig. 7a). We considered different demand distributions with either mostly short-range (the more proximate the nodes, the higher the demand between them) or mostly long-range (demand is higher between distant nodes) flows. Changing the demand-distribution from short-range to long-range, reduces the role of congested intra-community links and increases the role of congested inter-community links in slowing down network flows. We calculated the criticality score of links with different flow-demand scenarios and observed that altering the distribution of the demand, changes the link criticality scores as expected; this suggests that the criticality score index is consistent with the ground-truth (see Fig. 7c).

Action: We have now added a completely different and far superior accuracy-assessment analysis to the revised *Main Text*. The new analysis is performed on RGG networks with random link congestion and three different demand scenarios, as now explained in the section "Bottleneck identification." For practicality of computations in this analysis we chose the size of RGG networks as $n = 100$ with approximately 350 links. (For RGGs of size $n = 100$ nodes spread over the $[0, L]^2$ plane with $L = 10$, we chose the neighborhood radius of $r_0 = 1.5$ to ensure connectivity, and also have a large-enough set of links according to the RGGs' expected average degree of $(\pi r_0^2 n)/L^2 \approx 7.06$ [arXiv:0706.1063v2].)

In the first step, we found the "true ranking" of critical links by perturbing network links individually one by one, while determining each link's effect on the network reliability. Let α be the demand-serving reliability of the original network. Each time we picked a single link e_{ij} and after slightly increasing its quality by $q_{ij} + \varepsilon$ (we used $\varepsilon = 0.01$), we calculated the resulting improvement $\alpha' - \alpha$ as the true value of the link for the network's reliability, where α' is the reliability of the network after the perturbation in the link's quality. The perturbed link quality was set back to its original value before calculating the improvement effect of the next link. Network links were then ranked in descending order of their improvement effect on network reliability α. We were then able to choose the k top links in this ranking (where k was specified in advance) to obtain the true network bottlenecks; we denote this bottleneck set by TR.

To directly assess the accuracy of the proposed link "criticality score", we compared the ranking of links based on Criticality Score (CS) with the true ranking (TR) of links. We also compared a randomly shuffled ranking (as baseline) and the ranking based on Edge Betweenness centrality (EB), with the true ranking. To compare each ranking with the true ranking, we took the set of k top-ranked links in both rankings and counted the number of common links between the sets; we started by setting $k = 1$ and then gradually increased it.

Figure R1 (Fig. 4 in the revised manuscript) demonstrates the results of this experiment. Each curve is the result of 500 independent realizations of the RGG network with a particular random organization of link congestion and one of the three demand scenarios (i.e., short-range, homogenous, and long-range flow-demand). The set of top CS bottlenecks was found to be almost exactly the same as the set of top true bottlenecks (TR) with 98-100% of the elements matching for different k values; Fig. R1 shows the result for k values up to 150 which is less than half of the network links. The EB index was unable to achieve a comparable accuracy to that of our proposed CS index.

Note that the usefulness of the link criticality score index is that it can be calculated for all network links via efficient/scalable algorithms, e.g., our suggested modified Dijkstra's algorithm (see SI Note 3). However, finding the true ranking of links is only achievable through impractical (especially for large networks) approaches such as the brute-force search used here (which requires permuting a single link's quality by a predetermined value, calculating the network reliability, and repeating this for each single link on the network).

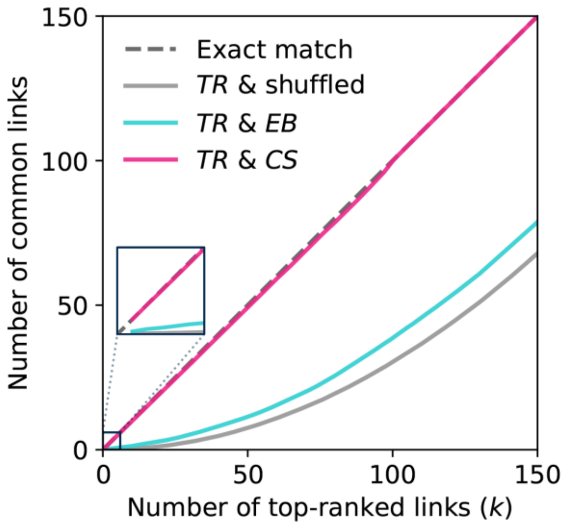

Fig. R1. Accuracy assessment of link criticality score index.

In summary, we assessed the accuracy of our proposed criticality score (s) index, by comparing the ranking of links based on criticality scores with the true ranking. When we picked equal-sized sets of top links in both rankings, they matched almost exactly (98-100% of the elements were in common to both sets). These results are now reported in the revised manuscript (see the section “Bottleneck identification” and Fig. 4).

2.2 Is there a ground-truth data for the most critical transportation links?

Response: The best ground-truth data that we found, provide a list of the most critical red spots or congestion “pain points” in Melbourne’s urban road network [https://www.redspotsurvey.com.au/]. This data is compiled by the RACV (Royal Automotive Club of Victoria), which is a major public company in the Australian State of Victoria. The analysis is performed based on both traffic data and data from surveying users. They reported 10 pain points (congested road locations significantly affecting traveling flows) in Melbourne’s network, but in fact half of these were not on a public transport corridor and thus could not be a part of our analysis. Among the remaining pain points, 4 out of 5 correspond to the links among the top 100 (<1%) bottlenecks found by our analysis.

Action: We had mentioned the above RACV report only briefly, but we have now elaborated and highlighted this in the revised version of the manuscript (in section “Bottlenecks of real transportation networks”).

2.3 Can authors argue that the improvement of transportation links according to the link criticality score is more efficient than that according to alternative measures, say, betweenness centrality?

Response: In a nutshell: **i)** No existing index is as specialized or as comprehensive as the proposed link criticality score for the purpose of measuring the effect of link congestion on flows in demand-serving networks. The criticality score accounts for network structure, link congestion, and demand distribution, while others do not. Also, we have performed a good deal of experiments and **ii)** demonstrated the superior performance of criticality score for network improvement, both in terms of increasing the proposed network reliability α and decreasing the passenger travel times. (See our section “Bottleneck amelioration” in the *Main Text*.) Finally, motivated by the reviewer’s comment we revised the manuscript and **iii)** directly showed with a new test that the criticality score index is able to accurately identify the true bottleneck links of networks (as presented in response to comment 2.1).

The results in our original manuscript and the new direct assessment of accuracy, all confirm that improving the network through links identified by the “criticality score” is very effective and in particular, significantly more effective than doing so according to the well-established structural measure of edge betweenness centrality.

Action: The new test (as explained in full detail in response to comment 2.1) and its results are now reported in the *Main Text* of the revised manuscript, which together with our pre-existing tests demonstrate the superior effectiveness of the proposed bottleneck links for network reliability.

On the technical level:

3. The quality attribute q_ij depends on travel times along link ij, which are determined by congestion levels and should strongly depend on the network structure. When one performs percolation, network links are removed and travel time along each surviving link should be updated. Since quality attribute values are functions of these times, should not quality attributes be updated at each step of the percolation procedure?

I invite authors to clarify this aspect in the main text. In case, this question has already been answered in previous studies, I invite the authors to provide relevant references.

We thank the reviewer for bringing this important potential source of confusion to our attention. We were always conscious about explaining this carefully, but now have made sure this is completely clarified in the revised manuscript.

Firstly, it is important to note that in the case of on-road Public Transportation (PT) networks, the link quality q_{ij} on any link e_{ij} is independent of the rest of the PT network’s structure and the passenger flow demand and it is only determined by the level of congestion on “road segments” connecting node i to node j. Nevertheless, below we explain why quality attributes are not updated at each step of the percolation by clarifying our usage of percolation model with references to previous studies, as the reviewer has asked.

In short, the percolation process (the whole process of link removal) is applied at each snapshot in time to unpack and characterize the organization of different levels of congestion on the network, and monitor this as it changes over a day. For each snapshot, the link qualities correspond to a particular time t and remain fixed during the percolation procedure.

To clarify our percolation analysis, we may divide the common percolation-based approaches for analysis of physical networks into two distinct classes, based on what the percolation process represents/studies. The first class of percolation processes, which we briefly mention in the “Introduction” section, simulate node/link failure and broadly speaking, aim at studying network “resilience” (or “robustness”). In this class of analyses, removed links (randomly or according to link attributes) are deemed to have undergone complete failure, and with each failure occurring indeed the network has changed and this often motivates updating the state of surviving links for the next percolation step (which may trigger more failures); e.g., [Sci. Adv. 2017: 3 (12) e1701079]. However, our approach belongs to the second class, where given the network and the state (i.e., quality) of its links at a single snapshot in time, we study the congestion on the network through a percolation analysis. Each link’s quality q_{ij} remains fixed during the whole percolation process at any snapshot in time. (Note though, over a day the quality of each link can vary between different snapshots in time.) Percolation process corresponds to the threshold ρ varying in $[0,1]$, and the process is monitored by UD_{ρ} which characterizes the way congestion is organized with respect to the distribution of flow-demand over a network snapshot. Comparing the network’s percolation properties, observed

separately at different snapshots, reveals the evolution of network's global properties over time (e.g., Fig. 5a,c according to the existing works and Fig. 5b,d using our proposed analysis). Highly respected research studies that use the same class of percolation analysis as ours, include [PNAS 2015: 112 (3) 669-672] and [PNAS 2019: 116 (1) 23-28], which both study transportation networks. Also, a study such as [PNAS 2012: 109 (8) 2825-2830] on brain networks is essentially studying the organization of link strength using percolation process on one snapshot of the brain network and belongs to this same category; the modular organization of link strength is being unpacked and there is no need for updating the remaining links when others are removed from the network.

Action: We had already explained that we are interested in studying how the continuous degradation of links (e.g., due to congestion) affects network performance, as opposed to how complete failure of links breaks down the network. (The complete link failure is a simpler phenomenon, more studied in the past, and it rarely occurs in reality compared to variation in link congestion.) Now, we have emphasized this further and highlighted the difference between using the percolation to simulate link/node failures and our usage to unpack the structural and dynamical properties of the network at a single snapshot in time. Recent major works in the literature that use the same percolation paradigm as ours, are directly pointed out in our explanations and we explicitly mentioned why link qualities remain fixed during the percolation process on one network snapshot. (Important revisions regarding this comment appear in the "Introduction" and "Unaffected demand and network reliability" sections of the revised manuscript.)

----

4. I invite authors to extend the analysis to cities other than Melbourne. In the current form, the manuscript is only analyzing traffic in one city and it is not clear how general the findings are. Tests on synthetic random geometric graphs only partially resolve the problem due to the uniform traffic patterns.

Response: This is a valid point, and we have always been aware that adding results from other cities helps clarify the generality of our findings, but as pervasive detailed travel records can be considered sensitive information, it is of course very difficult to get access to such information. However, this comment motivated us to persist in seeking for data of other networks, and fortunately our colleagues at University of Queensland kindly helped us by running our codes on one month of smartcard data from the city of Brisbane, Australia. We thank the reviewer for motivating the addition of results from another city to our manuscript, which we believe have enhanced the impact of this study.

Action: We have applied our proposed percolation-based analysis on the on-road (bus) public transportation (PT) network of Brisbane and added the results to the revised version of the manuscript. The network and its passenger flow demand are derived from detailed smartcard data (some 15 million transaction records) collected over one month (March 2013). Brisbane's bus network has an average size of 1400 (650) nodes connected via 3400 (1500) links during different times of a regular weekday (weekday). The network is serving the demand for about 250,000 (70,000) passenger Origin-Destination (O-D) trips over a weekday (weekend day). Figure R2a shows a snapshot of the network at 8:00 a.m. on a regular weekday, where link colors indicate the relative velocity of transport (link quality q). We calculated the network reliability indicators, namely, percolation criticality ρ_c and demand-serving reliability α, at different times over one month of data; see Fig. R2b,c for daily evolution of these indices averaged over different days, separately for weekdays and weekends.

In summary, the observations made in the results from Brisbane were consistent with the findings from application of our proposed framework to Melbourne's network. The reliability measure α captured a clear pattern in daily evolution of global network properties with indications such as reliability declining during peak hours, which suggest that the measure is reflecting the actuality. But the state-of-the-art measure ρ_c had relatively large fluctuations over the day, and there appeared to be no repeating pattern on a day to day comparison (see Fig. R2b,c). This was the same conclusion we reached from studying the Melbourne network. In addition, α successfully captured clear and distinctive patterns in global network dynamics during weekdays and weekends (Fig. R2c). (We have also discussed the differences between the reliability of the PT networks of Melbourne and Brisbane at the end of the section "Application to public transportation networks" of the *Main Text*.)

Figure R3a depicts the spatial distribution of overall link criticality score (suggested by our study) on Brisbane's PT network during weekdays. The network bottlenecks found by link criticality score were found around urban hotspots and important travel corridors in Brisbane. Similar to our experiments on Melbourne's PT network, we have simulated the amelioration of 2% of bottleneck links identified by different measures, namely, percolation criticality (PC), edge betweenness centrality (EB), demand-weighted edge betweenness centrality (WEB), and the proposed link criticality score (CS). Amelioration of CS bottlenecks compared to other competing methods, resulted in a larger reliability improvement consistently at different times of the day for both weekdays and weekends (see Fig. R3b,c). We have shown the new results in Fig. 2b and Fig. 5c,d of the *Main Text* and Fig. S10 of the *SI*, and discussed them in the *Main Text*.

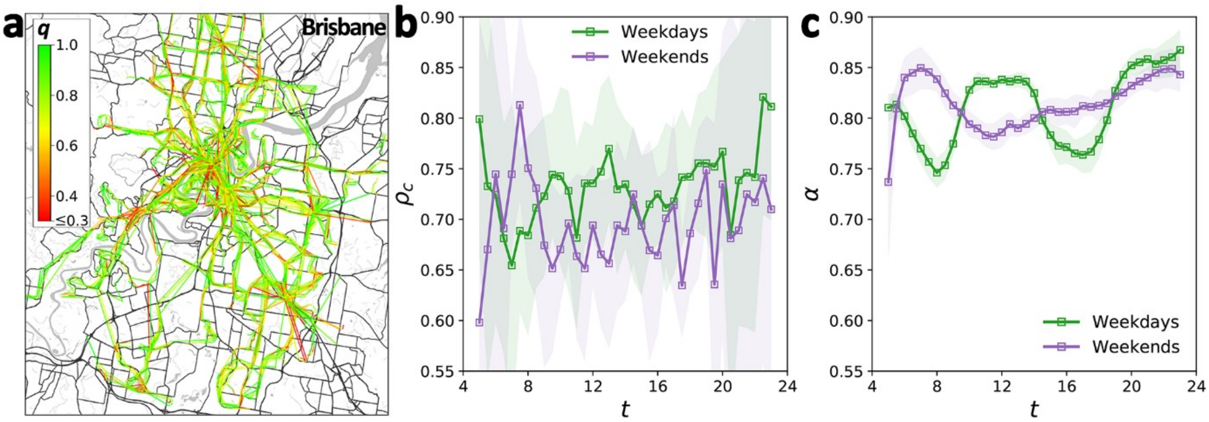

Fig. R2. Reliability analysis of Brisbane's on-road public transportation (PT) network. **a** One snapshot of Brisbane's PT network, taken at 8:00 on 1 March 2013 (weekday), with color-coded link qualities (q_{ij}). **b,c** Daily evolution of percolation criticality ρ_c (b) and demand-serving reliability α (c) of the network over a day. Lines show the average over different days and the shaded areas indicate the standard deviation from the average calculated separately over weekdays and weekends.

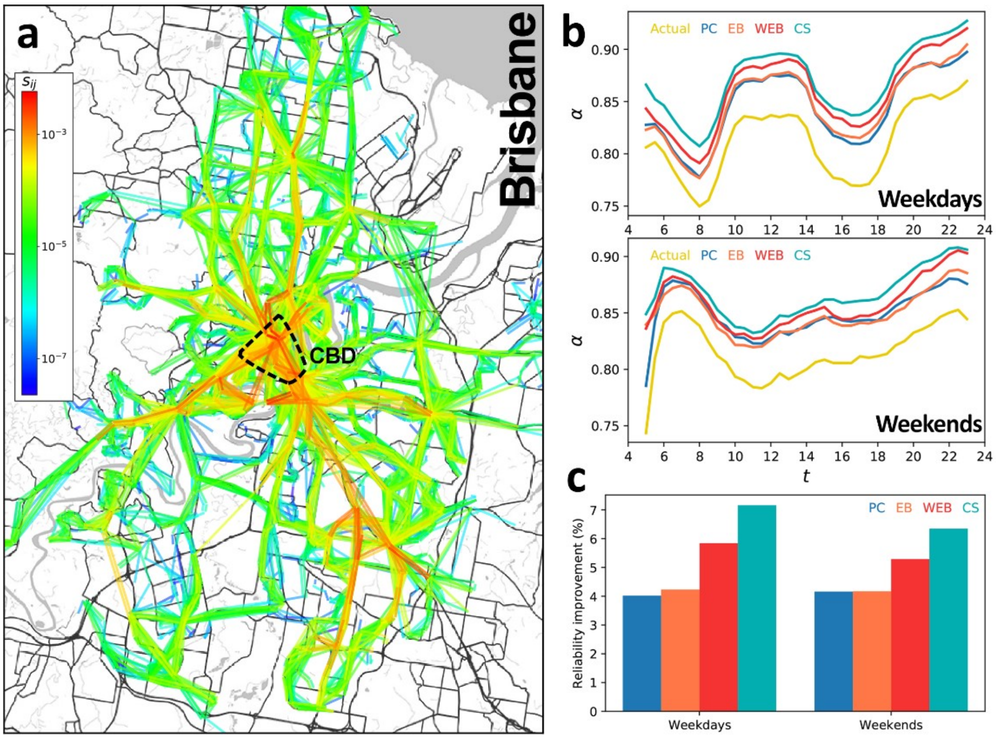

Fig. R3. Improving network reliability, through bottleneck amelioration. **a** Map of overall link criticality scores in Brisbane. **b** Average network reliability (α) improvement over the day, by amelioration of different types of bottlenecks. **c** Comparison between different bottleneck identification methods, in terms of their improvement effect on network reliability.

----

5. When modeling random geometric graphs, the authors set the connectivity parameter of r_0 to 1.5. How is this parameter selected?

Response: We are glad that the reviewer pointed this out, and unfortunately, it appears we made a minor mistake by not updating the text to the correct RGG settings that corresponded to our latest results. The presented experiments in the section "Generality of the proposed framework" are performed on RGG networks of size $n = 2500$ spread over the space $[0, 50]^2$, and links connecting any of nodes with distance less than r_0, while we had set $r_0 = 1.6$ to ensure connectivity in the resulting RGGs. (The parameter r_0, sometimes called "neighborhood radius," essentially determines the extent to which node-pairs should be connected via links when generating an RGG structure, thus it directly affects the connectivity and average degree of these networks.) It has been shown [M. Penrose 2003: random geometric graphs; arXiv:cs/0702074] that there exists a critical threshold for the radius $r_c \approx L.\sqrt{\ln(n)/(\pi n)}$ for which an RGG graph with $r_0 > r_c$ is asymptotically almost surely connected.

With network size $n = 2500$ spread on a square of edge-length $L = 50$, we have $r_c \approx 1.57$. Thus, we chose $r_0 = 1.6$ which almost guarantees that the resulting RGGs are connected (we also checked if each generated network is connected) while not being too large so that the network loses its structural property of having 'bridged local communities'.

We have added a new experiment on RGG networks of size $n = 100$ with nodes spread within $[0{,}10]$, for which the connectivity threshold is at $r_c \approx 1.21$ but we chose $r_0 = 1.5$ not only to ensure structural connectivity but also to have the desired number of links on the network.

Note that our experiments on RGG networks make perfect sense only if the network is connected, and other than that r_0 should be large enough not to allow isolated nodes or

components (or maybe not too large so that the network would become a clique), the choice of r_0 does not change the key conclusions reached from our experiments.

Action: We have added a clear explanation with referencing, on how we chose the value of r_0 for constructing the RGG structures in the revised manuscript.

In summary, the present manuscript contains interesting findings that might have significant impact on our understanding of transportation efficiency. Since this impact is not sufficiently well established, I cannot recommend the publication of the manuscript in Nature Communications in its present form. A careful revision of the manuscript according to my comments might make the manuscript publishable in the future. My recommendation stands as “revise and resubmit”.

We have carefully revised the manuscript according to each comment and hope that it has reached a satisfactory level of clarity.

Reviewer #2 (Remarks to the Author):

This study represents a very good interdisciplinary research of transportation science and network science. The match between traffic demand and network percolation is of great concern for traffic management. However, considering the complexity of large transportation networks, the lack of effective theories and techniques is the “pain point”. Based on real smart-card data, the authors applied a percolation-based method to analyze the public transportation reliability of a typical infrastructure network – the public transportation system in Melbourne. The authors introduced a demand-serving reliability (i.e., based on Unaffected Demand) which is the combination of flow demand and traffic percolation. Within this framework, the road affecting much the flow demand can be viewed as the “bottleneck”. Furthermore, the authors uncovered how the identified bottlenecks can improve the whole traffic.

Percolation theory has recently been widely applied to analyze the properties of the traffic network. However, to the best of my knowledge, this work is original and interesting to apply in the real flow demand in traffic percolation. The authors’ definition of demand-serving reliability is very insightful and practical for traffic management.

Based on their framework, they build up a tie between the microscopic link importance (bottlenecks) and the macroscopic system performance (reliability) in a simple but effective way as manifested by Eq. (5), which is one of their key results. Indeed, improvement of a few bottlenecks identified by their methods can significantly influence the global network performance, which is demonstrated by real data testing. It is an interesting perspective to consider the relationship between flow distribution and its traffic percolation process. With implications in both model networks and real-world networks, the authors have demonstrated that the uniformly distributed flow network shows similar percolation properties to the classic percolation model. This finding can shed light on future traffic management strategies – like by adjusting the locations of city functional regions to generate more OD paths with short distance and large flow.

Considering its novel and noteworthy findings, as well as the significant contributions it makes to the related fields, I believe that this paper fits perfectly the publication standard required by Nature Communications.

We thank the reviewer for the positive and useful comments, which have led to improvement of the presentation and clarification of our proposed analysis.

Before publication, I would like to ask the authors to clarify some of the following points:

(1) The authors discuss well the percolation of public transportation. In this sense, I wonder if the multiple paths identified by the authors between the same pair can be compared in time scale. This could of course influence the travel experience of each user.

Response: Our proposed analysis works with the state-of-the-art traffic percolation (as in, e.g., [PNAS 2015: 112 (3) 669-672] and [PNAS 2019: 116 (1) 23-28]). Involving the path travel times is an interesting problem and we had thought of using it to compare multiple paths between each origin-destination (O-D) pair. Although traffic percolation is based on "link" travel times, working with "path" travel times proves to be very difficult within the percolation-based framework. This is because after removal of a link with a certain level of congestion, looking at the remaining paths does not show the effect of that link's "congestion" on travel times, at least not in the present model. The model would have to be significantly extended to allow for this, and we believe the analysis would become unwieldy. Comparing multiple paths between each node-pair in time scale also requires to involve the passenger route choice behavior to the analysis. In many ways this falls outside the scope of this paper.

Nevertheless, we explored path travel-time where we could, specifically in the section "Bottleneck amelioration" but from a different direction. In that section we considered the shortest travel-time path as the primary path chosen by passengers between each O-D pair and we demonstrated how ameliorating the congestion on our identified bottlenecks (which reduce the conflict between flows and congestion) leads to significant reduction in travel times over the network.

Action: We had already discussed the path travel time with respect to amelioration of bottleneck links. In the "Conclusion" section of the revised manuscript we have indicated the complexity of the analysis when studying travel time and why it cannot be done via existing percolation models, and that there is much needed work to be done in this area.

----

(2) Following the above question, when a given path is destroyed, can the alternative paths between the same OD pair match the amount of total traffic demand? Will some paths left influence the cascading failure effect?

Response: We thank the reviewer for bringing to our attention that we need to clarify this point better in the manuscript.

Firstly, we did not directly take into account links' passenger capacity for a number of reasons (which will become clear shortly) but followed the methods of existing seminal works in the literature. In the *Appendix A* at the end of this document (the reviewer may skip those details without loss of continuity) we show how our work is identical at the conceptual level to the state-of-the-art approaches, where capacity is also not considered an issue.

Motivated by the reviewer's comment, however, we calculated the capacity of the network under percolation to check whether it could match the amount of travel demand. Here we define capacity as the maximum proportion of the total passenger travel demand that can be moved between Origin-Destination (O-D) nodes over the network of a particular time. The method used for calculating capacity at any value of ρ is described in *Appendix B* (of this

response letter) and copied in the *SI* as well. The calculation is based on the maximum number of passengers that can be carried on network links according to the number of Public Transportation (PT) vehicles running on each link and the maximum capacity of these vehicles. We plot the capacity in red in Fig. R4; the figure shows the same percolation process as in Fig. 2d which is performed on a network snapshot at 8:00 a.m. on a weekday. All units are relative to the total flow-demand on the network when no link is deleted yet. (In other words, the reference total flow demand is the unaffected demand when $\rho = 0$, i.e., $UD(0)$.) According to our scheme, when $\rho = 0$, the unaffected demand on the network is $UD(0) = 1$ while the actual passenger capacity of the network (that we have calculated according to *Appendix B*) is $C(0) = 2.75$ larger and thus not at all limiting. Note that the total capacity (red) decreases with ρ because there are less pathways to take passengers as links with quality below ρ (as some have been deleted).

The results in Fig.R4 show that the flow-capacity of real PT networks (Melbourne and Brisbane) during our percolation procedures always remains above the amount of flow that corresponds to the UD; the network under percolation can always handle 1.5 to 3 times the proportion $UD(\rho)$ of the total demand on the network. This means that there is always sufficient capacity for carrying the volume of flows on alternative pathways between connected O-D pairs. The figure (Fig. R4) portrays the capacity of Melbourne's PT network at the morning rush-hour on a weekday where the demand is at its maximum.

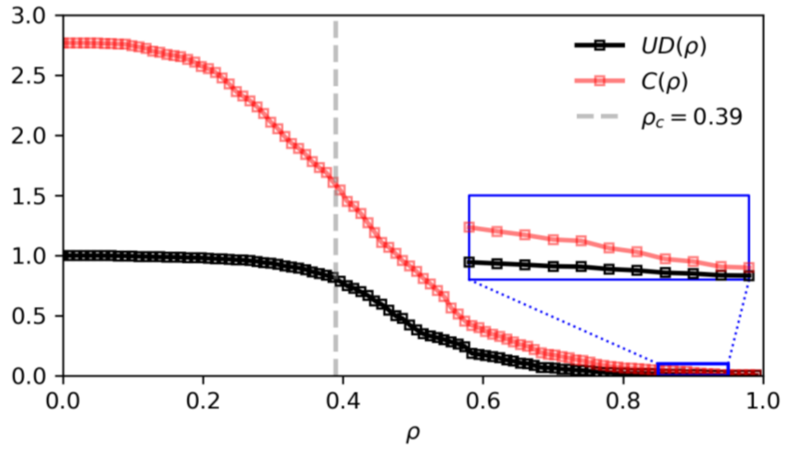

Fig. R4. Percolation process on a snapshot of the Melbourne's PT network monitored by $UD(\rho)$ (same as in Fig. 2d of the *Main Text*), and additionally including the capacity $C(\rho)$ plotted in red.

See the *SI* Note 2 of the revised manuscript for description and the result of these calculations and discussion on the capacity of the links in reliability analysis.

We would like to add an important point, as to why we believe that our proposed framework provides a valuable analysis without considering whether or not the remaining pathway can match the amount of flow between O-D pairs. In on-road public transportation networks which are the focus of our real-world analysis, link passenger capacities are determined by the capacity of the bus/tram vehicles and the number of services which are almost independent of level of congestion on network links. If a PT vehicle is upgraded to have more capacity or even if a new PT service is added to the network, the congestion on roads remains fixed (or barely changes) but this increases the passenger capacity of all the links that the vehicle runs through. So, our proposed UD can be used to pinpoint the issues on the network such as critical bottleneck links which if improved effectively minimize the conflict between passenger flows and congestion, but then if there are any shortcomings related to passenger capacity of links they can be attended completely in parallel or independently (as mentioned, for example, by adding a PT service to the link or upgrading the PT vehicles to have more capacity).

On the cascade failure effect. The reviewer has asked about the cascade failure effect. Note that our percolation model is similar to the state-of-the-art traffic percolation framework (e.g., in [PNAS 2015: 112 (3) 669-672]), where the percolation process is performed on "a single network snapshot in time" to study the organization of congestion rather than simulating failures and the network's reaction to failures over time. Nevertheless, in on-road public transportation networks, even if some links fail completely on the actual network, the addition of passengers to the functional links does not trigger new failures because even if passengers waiting for a service grow more than the capacity of a bus or tram, they will be carried by the next vehicle(s) or worst case will not be carried at all. (Unlike road traffic networks, additional load does not cause the remaining services/links in on-road PT networks to fail.)

Action: We have reported the results presented in the above response, with more technical details on the capacity calculations in the *SI* Note 2 of the revised manuscript. The new section also describes a possible capacity-aware extension of our analysis and its implications.

----

(3) In Fig. 2, authors claim that at percolation critical point, the unaffected demand is still 80%. Is this calculation consider the flow capacity of the giant component?

Response: If this comment is concerned with capacity of the network links for unaffected demand (UD) at percolation critical point, the response to the previous comment and Fig. R4 (where criticality is marked by a dashed gray line) covers this as well. However, we think that the reviewer is concerned with how flows on the giant component (GC) relate to that 80% of the total demand which remains unaffected after the percolation criticality. For simplicity, let us imagine a network for which the total demand is fully contained on the GC (thus UD=100%) with this going through no change until the percolation critical point. And at the percolation threshold ρ_c, the GC suddenly fragments into multiple isolated components and UD drops to 80%. The UD tells us that 20% of the network demand was between origin-destination node pairs for which the two ends were placed on different isolated components (which emerged as a result of GC fragmentation). In other words, this 80% of the demand includes the flows which were on the GC right before the criticality and also can remain on the isolated components resulting from fragmentation of the GC, but the previous flows from one isolated component to another isolated component are not counted in this (80%) proportion of the demand.

Action: We have now carefully revised the explanations regarding the calculation of UD (especially those directly related to the example in Fig. 2d) and have made sure of their clarity.

----

(4) The traffic reliability alpha is found larger in weekdays than in weekends as shown in FIG. 2e. The authors argue that this may be due to the impact "by lower-quality links during weekends compared to weekdays". Can the authors give some examples of specific roads? Because when comparing the spatial distribution of critical links (as shown in FIG. S8A), it looks very similar between weekdays and weekends to readers.

Response: We have explained this from a different perspective which we believed has clarified the point in the revised manuscript, and below is our explanation in response to the comment.

The on-road (bus/tram) PT system in Melbourne is planned differently for weekdays as compared to weekends. In particular, there are many more services during weekdays and as a result not only does the system have a much higher capacity, but the network representation of the system has many more links compared to the weekends' network. The lower reliability of Melbourne's network during weekends is thus mainly attributed to the network having much

lower link density (weaker connectivity) due to the greatly reduced number of services, compared to weekdays (the network is fine-tuned for weekday demand). As seen in Fig. R5, the number of links increases fourfold from weekend to weekday.

In the argument quoted in the comment, we are merely explaining how UD measurement views the difference between weekdays and weekends network. To describe the difference from UD's point of view we had it written: "a larger proportion of the demand has to pass through lower-quality links during weekends compared to weekdays." This means that if we take lower-quality links (highly congested links, say, $q_{ij} < 0.3$) on two networks, one from weekdays and another from weekends, the demand distribution on the two networks is such that a larger proportion of the flow-demand has to pass through the lower-quality links on the weekend networks. So, it was not meant that some specific links are doing worse over the weekend, but more about how the demand and congestion are distributed with respect to one another.

As the reviewer asked for a specific example, we made a check on Melbourne's network data and observed that: a larger proportion of the flow-demand is directed to the congested central business district (CBD) of Melbourne over the weekends. We can explain why this is consistent with what we are arguing about the difference between the weekdays and weekends. Melbourne is a monocentric city with almost a square-shaped (2 km^2) CBD in the middle of the metropolitan area while congestion is concentrated inside and around this area most of the times. We approximated the CBD area by a circle with the radius of 1 km centered at a point in the middle of this CBD area. The proportion of the network flow-demand that originates from outside this circle and ends inside it is approximately 13% at weekday rush-hour (8:00 AM) but this proportion is larger and between 18-19% at different times over a regular weekend.

It is worth mentioning that in Fig. 8A the spatial distribution of critical links is shown for all links appearing on networks of weekdays and weekends at different times. As both weekdays' and weekends' maps show an immensely large number of links with concentration of critical links around the CBD area, it is difficult to differentiate the details, but a closer look shows i) the higher link density of the network on weekdays and ii) the higher criticality of links inside, north, and east of the CBD area on weekends. Both these factors along with the different demand distribution have a role in lowering the reliability of the Melbourne weekend network. Please also refer to the next response (for comment (5)) for further elaboration.

Action: In the section "Application to public transportation networks" of the *Main Text*, using the above explanations, we have clarified that the network structures are very different between weekdays and weekends, and presented the main factors that lead to lower reliability of weekends' networks.

----

(5) In line 70-71, the authors tell us the total flow demand of on-road PT in weekdays and weekends, where a big volume gap exists (470,000 - 210,000 = 260,000 > 210,000). Generally, if the flow demand increases so much, the network reliability could have hard time to stay in a higher level. However, the results show that in weekends the network reliability is much lower than in weekdays. The effectiveness of the reliability measure may depend on how much the PT takes up in the whole on-road transportation system. Can the authors discuss and clarify this issue?

Response: We thank the reviewer again for the careful examination of the manuscript. The answer to this is actually straightforward. The difference between the passenger volume is of course true, but as previously mentioned, the on-road (bus/tram) PT system in Melbourne is

planned extremely differently between weekdays and weekends. In particular, there are many more services during weekdays, and as a result not only does the system have a much higher capacity, but the network representation of the system has many more links compared to the weekends' network. As seen in Fig. R5, the number of links increases fourfold from weekend to weekday. The explains why weekday networks can handle the increase in passenger volume (as the reviewer mentioned by 470,000 - 210,000 = 260,000 trips). (Of course, this suggests that the network planners had considered the discrepancy between the passenger volumes and allocated much more resources to run the network on weekdays.)

Regarding the reliability of the network, as we have mentioned, weekday networks have much higher link density, making the structure of the network stronger from a connectivity point of view; see Fig. S7B in the SI showing the larger average degree of weekday networks. Higher link density on weekdays means the availability of more paths between nodes; this means that if a path is ruptured by highly congested links generally it is more likely that an alternative path exists. Lower link density of the network on weekends, and simultaneously a larger proportion of the passengers to travel to/from hotspot areas with more congestion, results in more conflict between flows and congestion (which is what our reliability index α measures) and thus leads to a lower α. Note that the reliability α can be useful to compare different networks. For example, lower overall reliability α of Melbourne's PT network during weekends shows that there is more room for improvement in weekends' networks compared to weekdays' by, say, separating bus lanes on weekends' network bottleneck links. (It is worth mentioning that we added the same analysis on bus network of Brisbane to the paper, and there although the daily evolution of reliability α differed between weekend and weekdays, yet unlike Melbourne, α was evolving within approximately the same range of values in both weekdays and weekends.)

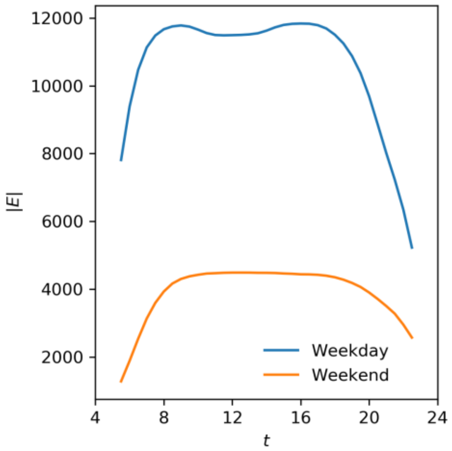

Fig. R5. Average number of links $|E|$ versus time t in weekday and weekday networks in Melbourne.

Action: We have added Fig. R5 as a subplot to Fig. S7 in the *SI* and discussed it in the *Main Text* to clarify the extremely different PT network design of weekdays versus weekends in Melbourne. We have now highlighted the extreme difference in link density (connectivity) and number of links (capacity) of Melbourne's PT networks between weekdays and weekends, which allows the weekday network to contain the large passenger volume and even helping the reliability of the network by strengthening the structure from a connectivity perspective (see the section "Application to public transportation networks").

Appendix

A. Similarity of our approach to well-known traffic percolation analyses in terms of independence from capacity. Our approach is similar to that found in state-of-the-art traffic percolation analyses, e.g., [PNAS 2015: 112 (3) 669-672] and [PNAS 2019: 116 (1) 23-28], firstly, in the sense that the whole process (of the percolation simulation) is performed on the network at a single "snapshot" in time with all links being actually functional but at different congestion levels. Here, percolation can be viewed as a "hypothetical" link removal process which unpacks the organization of congestion on network structure. Secondly, this type of analysis does not pay attention to the load and capacity of links, and as a result the analysis does not conclude anything about the problems with capacity in the network. Loosely speaking, following the above principles the existing traffic percolation ([PNAS 2015: 112 (3) 669-672]) studies the relative speed (inverse of congestion level) at which "an individual passenger" can travel most (giant component) of the network. At a fundamental level, our framework follows all the above principles, but in addition, considers that "an individual passenger" is more likely to travel between Origin-Destination (O-D) points with higher demand; the likelihood of a trip between different O-D points is in proportion to the distribution of the demand over the network. So, in short, both our proposed framework and the well-established traffic percolation analyses (referenced above) unpack the organization of congestion on one network snapshot and do not check for capacity problems. They both do not conclude anything about capacity issues but can pinpoint the problems with organization of congestion over the network structure.

B. Calculating the maximum flow-capacity of a demand-serving network. Using the notation of the manuscript, We assumed a maximum capacity of 50 passengers per bus/tram (conservative choice) to assign a flow-capacity to each network link, and then at each threshold ρ in the percolation process we approximated the capacity of the subnetwork G_ρ by solving a "maximum concurrent multicommodity flow" problem using Fleischer's algorithm [SIAM J. Discrete Math 2000: 13 (4) 505-520]. To explain in short how the capacity is calculated here, note that the important implication of the above the problem is considering the relative amount of demand between each pair of nodes and that they should be served concurrently. Now, take a very small λ (close to zero) so that the network has the capacity for concurrent movement of $\lambda . r_{od}^{\rho} . f_{od}$ passengers between all (o, d) node-pairs. For any subnetwork G_ρ $(\rho > 0)$ during the percolation, Fleischer's algorithm gradually increases λ to find the largest possible λ for which simultaneous movement of $\lambda . r_{od}^{\rho} . f_{od}$ units of flow between all (o, d) pairs is possible considering the capacity of network links; let us denote the maximum value of λ found by the algorithm as $\lambda_{max}(\rho)$. If $\lambda_{max}(\rho) \geq 1$ it means that the network at threshold ρ has the capacity for the demand between all pairs that are still reachable, otherwise the network can accommodate the proportion $\lambda_{max}(\rho)$ of that demand. Maximum concurrent flow-capacity of a subnetwork G_ρ, normalized by the total demand on the actual network G_0 can be calculated as $C(\rho) = \lambda_{max}(\rho). UD(\rho)$.

If a demand-serving network functions close to its flow-capacity and one wants to study the problems with capacity of the network in addition to the conflict between flows and congestion, then, our definition of UD can be extended to $UD_c(\rho) = min\{1, \lambda_{max}(\rho)\}. UD(\rho)$. The new capacity-aware unaffected demand $UD_c(\rho)$, monitors the proportion of the total demand that can be "accommodated" between O-D pairs only on links with quality above the threshold ρ; during the percolation always $UD_c(\rho) \leq UD(\rho)$, and $UD_c(\rho) < UD(\rho)$ if O-D paths on G_ρ cannot match the remaining demand over the subnetwork. Accordingly, the reliability measure α can be extended to capacity-aware reliability α_c defined as the area under the curve of $UD_c(\rho)$ over $\rho \in [0,1]$.

Reviewer #1 (Remarks to the Author):

My concerns were addressed in full in the revised version of the manuscript and now I can conditionally recommend the manuscript for publication.

I note that the manuscript has undergone substantial changes, one which is related to the inclusion of a new city into the analysis. As a result, some changes were made in the main text, figures, and figure captions. I invite authors to carefully check the consistency of the manuscript wrt to these changes. In particular, the inclusion of Brisbane led to the addition of panel b into Fig.2. The caption of Fig.2, however, was not properly updated, some references to figure panels from the legend were not updated. Also, it is not clear which city panels (c) and (d) correspond to.

Reviewer #2 (Remarks to the Author):

I read carefully the authors' response letter to my comments and their revised manuscript and I highly believe that the authors answered all my concerns and the current version of the manuscript is suitable for publishing in Nature Communication.

Reviewer #1 (Remarks to the Author):

My concerns were addressed in full in the revised version of the manuscript and now I can conditionally recommend the manuscript for publication.

I note that the manuscript has undergone substantial changes, one which is related to the inclusion of a new city into the analysis. As a result, some changes were made in the main text, figures, and figure captions. I invite authors to carefully check the consistency of the manuscript wrt to these changes. In particular, the inclusion of Brisbane led to the addition of panel b into Fig.2. The caption of Fig.2, however, was not properly updated, some references to figure panels from the legend were not updated. Also, it is not clear which city panels (c) and (d) correspond to.

Authors' response: We thank the reviewer for their careful examination of the manuscript. We have fixed the typo in the caption of Fig. 2 (at some point subfigure **b** was called instead of subfigure **c**). Also, we revised the caption of Fig. 2 to make it clearer and directly mentioned that Fig. 2**c,d** correspond to the Melbourne's network visualized in Fig. 2**a**. We have carefully checked and made sure of the consistency of the manuscript with respect to the changes made during revision.

Reviewer #2 (Remarks to the Author):

I read carefully the authors' response letter to my comments and their revised manuscript and I highly believe that the authors answered all my concerns and the current version of the manuscript is suitable for publishing in Nature Communication.

Authors' response: We are pleased that all reviewer's concerns are addressed.
